# The Evolution of Next-Generation Glucose Monitoring Systems: Developments from Conventional Diagnostics to Wearable and Continuous Sensing Platforms—A Narrative Review

**DOI:** 10.3390/bios16090525

**Published:** 2026-09-20

**Authors:** Sumedha Nitin Prabhu

**Affiliations:** Biomedical Science & Engineering Programme, School of Medicine, The Chinese University of Hong Kong, Shenzhen 518172, China; sumedhanitinprabhu@cuhk.edu.cn

**Keywords:** diabetes diagnosis, glycemic control, glucose monitoring, finger-prick electrochemical glucometers, wearable sensors, continuous glucose monitoring, minimally invasive sensing, non-invasive sensing, electrochemical sensors, artificial intelligence-assisted wearable technology

## Abstract

For the diagnosis of diabetes, glycemic control, treatment modification, and the avoidance of both acute and long-term consequences, accurate glucose monitoring is crucial. This study examines the development of glucose-monitoring systems, from traditional diagnostic tests and finger-prick electrochemical glucometers to wearable, continuous, minimally invasive, and non-invasive sensing platforms. Major invasive technologies, including enzymatic and non-enzymatic electrochemical sensors, microdialysis systems, hexokinase-based photometric assays, and field-effect transistor biosensors, are critically evaluated. The ability of minimally invasive methods to access interstitial fluid with less pain is discussed, including fluorescence probes, surface-enhanced Raman spectroscopy, and microneedle arrays. Emerging non-invasive approaches are also evaluated. These include reverse iontophoresis, optical coherence tomography, infrared and photoacoustic spectroscopy, microwave resonators, smart contact lenses, and AI-assisted wearable systems. Finally, the review identifies major barriers related to sensitivity, selectivity, calibration, biofouling, biocompatibility, physiological variability, and clinical translation. It also outlines opportunities for intelligent, painless, and patient-centered glucose monitoring.

## 1. Introduction

Glucose is the dominant circulating carbohydrate fuel in humans and a central biochemical indicator of metabolic health. Blood glucose is maintained within a narrow physiological range by coordinated endocrine, neural, hepatic, renal, and peripheral-tissue mechanisms [1]. This regulatory network balances intestinal glucose absorption, hepatic glucose production, peripheral uptake, and renal handling [1]. According to World Health Organization (WHO) estimates, by 2027, it is estimated that there will be more than 2 million deaths from noncommunicable diseases like diabetes and kidney disease caused by diabetes [2]. For the majority of healthy persons, normal blood sugar levels should be between 4.0 and 5.4 mmol/L (72 to 99 mg/dL) when fasting, up to 7.8 mmol/L (140 mg/dL) 2 h after eating. Before meals, blood sugar levels should be between 4 and 7 mmol/L for those with type 1 or type 2 diabetes. For those with type 1 diabetes, post-meal levels should be less than 9 mmol/L, and for those with type 2 diabetes, the levels should be less than 8.5 mmol/L [3]. The sustained elevations above the normal range indicate impaired glucose regulation and may progress to diabetes mellitus. Diabetes mellitus, particularly type 2 diabetes, can cause a further complex cascade of diseases [4] in other organs, including the kidneys, eyes, the cardiovascular system, and nerves [5,6]. Glucose concentrations fluctuate in response to food intake, physical activity, stress, sleep, medication, and hormonal rhythms. Accurate assessment of these dynamics is therefore essential for both diagnosis and day-to-day therapeutic decision-making.

Diabetes mellitus has become one of the most significant chronic disease challenges of the twenty-first century. The global prevalence of diabetes has increased sharply over recent decades, driven by population aging, urbanization, sedentary lifestyles, obesity, and dietary transitions. The WHO and other international health agencies have repeatedly emphasized the growing burden of diabetes and its complications on individuals, health-care systems, and national economies. Poorly controlled hyperglycemia contributes to progressive vascular and neurological injury, leading to retinopathy, nephropathy, neuropathy, impaired wound healing, cardiovascular disease, stroke, and premature mortality [6]. Conversely, hypoglycemia, particularly in individuals receiving insulin or insulin secretagogues, can produce acute neuroglycopenic symptoms, seizures, coma, and, in severe cases, death [6]. Thus, both high and low glucose excursions are clinically consequential, and the ability to monitor glucose accurately, rapidly, and conveniently remains a cornerstone of effective diabetes management [7].

Traditional glucose assessment has relied on laboratory-based blood analysis, oral glucose tolerance testing, glycated hemoglobin measurement, and self-monitoring of blood glucose using finger-prick capillary samples. These methods have transformed diabetes diagnosis and care by enabling quantitative evaluation of glycemic status. However, each approach has important limitations. Laboratory assays provide high analytical accuracy but are episodic, require clinical infrastructure, and cannot capture short-term glycemic variability. Glycated hemoglobin reflects average glycemia over several weeks but provides limited information regarding daily glucose excursions, postprandial spikes, nocturnal hypoglycemia, or treatment-related variability. Finger-prick testing is portable and widely used, yet it remains invasive, painful, intermittent, and dependent on user compliance. As a result, conventional monitoring may fail to detect rapid glucose fluctuations and can provide an incomplete picture of the patient’s metabolic state. The inability of episodic measurements to resolve short-lived or asymptomatic glycemic excursions created a clear need for technologies capable of longitudinal glucose tracking. This unmet need drove the transition from discrete testing to continuous and wearable monitoring.

Continuous glucose monitoring systems have consequently transformed diabetes care by providing near-real-time measurements, trend information, and predictive alerts. Continuous glucose monitoring systems have changed diabetes care by providing near-real-time glucose readings, trend information, predictive alerts, and integration with insulin pumps, smartphones, and cloud-based digital health ecosystems. By measuring glucose in interstitial fluid through subcutaneous sensors, these systems allow patients and clinicians to evaluate glycemic variability more comprehensively than is possible with isolated finger-prick measurements. Continuous glucose monitoring has also enabled the development of hybrid and automated closed-loop insulin delivery systems, bringing diabetes care closer to precision and personalized medicine. Nevertheless, current commercial sensors still face challenges related to accuracy during rapid glucose changes, physiological lag between blood and interstitial glucose, calibration requirements, sensor drift, biofouling, skin irritation, limited wear time, cost, and accessibility. These unresolved issues continue to motivate research into more robust, comfortable, affordable, and fully non-invasive glucose monitoring technologies. Addressing these limitations requires more than improving a single sensor design. It requires a comparative understanding of how sampling route, biological matrix, recognition chemistry, and transduction mechanism jointly determine performance.

Accordingly, glucose-monitoring technologies can be classified along two complementary dimensions: the degree of tissue interaction required for analyte acquisition and the mechanism used for signal transduction. From a sampling perspective, glucose monitoring systems include invasive blood-based devices, minimally invasive interstitial fluid sensors, and non-invasive approaches that quantify glucose via the skin or in alternative biofluids such as sweat, saliva, tears, and breath condensate. From the perspective of signal transduction, electrochemical and optical methods have emerged as the two most extensively investigated families of glucose sensors [8,9]. Each class offers distinct advantages and limitations, and the continuing evolution of next-generation glucose monitoring is increasingly shaped by advances in materials science, nanotechnology, microfabrication, flexible electronics, biointerface engineering, wireless communication, and data analytics.

Glucose technologies can be classified along two complementary dimensions: the degree of tissue interaction required for analyte acquisition and the mechanism used for signal transduction. In this review, glucose sensing refers specifically to the physicochemical recognition of glucose and its conversion into an analytical signal, whereas glucose monitoring denotes the broader measurement system, encompassing sampling, sensing, calibration, signal processing, data transmission, display, and clinical interpretation. From a sampling perspective, invasive methods require blood collection or the insertion or implantation of a sensing component beneath the skin; minimally invasive methods cross the skin barrier with limited penetration or tissue disruption, as exemplified by microneedle and shallow interstitial fluid platforms; and non-invasive methods acquire glucose-related information without penetrating the skin or another epithelial barrier. Hybrid systems are classified by their most invasive component and explicitly identified where appropriate. From a transduction perspective, electrochemical and optical methods constitute the two most extensively investigated families of glucose sensors. This two-dimensional classification is applied consistently throughout the review.

Electrochemical glucose sensors remain the most mature and commercially successful technology for glucose monitoring. Their widespread use is attributed to their high sensitivity, rapid response, relatively simple instrumentation, low power consumption, compatibility with miniaturization, and low-cost manufacturing. Most electrochemical glucose sensors rely on enzymatic recognition, particularly glucose oxidase or glucose dehydrogenase (GDH), coupled with amperometric [10], potentiometric [11], impedimetric, or conductometric transduction. Amperometric sensors determine glucose concentration by measuring the current generated through oxidation or reduction reactions at a fixed applied potential [10]. Potentiometric sensors measure the potential difference between working and reference electrodes under negligible current flow. The working-electrode potential varies with analyte activity. Considerable progress has been achieved through the engineering of electrode materials, redox mediators, membranes, nanostructures, and immobilization strategies to improve analytical performance, including sensitivity, selectivity, detection limit, stability, and response time [12,13,14].

The development of nanostructured and functional materials has further accelerated progress in electrochemical glucose sensing. Carbon nanotubes, graphene, carbon black, metallic nanoparticles, conductive polymers, metal–organic frameworks, transition-metal oxides, MXenes, and hybrid nanocomposites have been explored to enhance electron transfer, increase electroactive surface area, improve enzyme loading, and reduce interference from electroactive species such as ascorbic acid, uric acid, and acetaminophen. In parallel, non-enzymatic electrochemical sensors based on noble metals, transition metals, and metal oxides [15] have attracted attention because they may overcome some limitations of enzymatic sensors, including enzyme instability, temperature dependence, pH sensitivity, and limited shelf life. However, non-enzymatic platforms often suffer from insufficient selectivity in complex biological fluids and require careful surface design to achieve clinically relevant performance. Although electrochemical platforms remain the most technologically mature, the requirement for direct contact with blood or interstitial fluid continues to motivate complementary sensing strategies. Optical methods address this need by interrogating glucose-dependent molecular or tissue signatures through photonic rather than exclusively electrochemical transduction.

These considerations have established optical sensing as a major research direction, particularly for minimally invasive and non-invasive glucose monitoring. Optical glucose sensing represents another major research direction, particularly for the development of non-invasive or minimally invasive monitoring systems. Optical approaches, including fluorescence [16], colorimetry, near-infrared spectroscopy, mid-infrared spectroscopy, Raman spectroscopy, surface-enhanced Raman spectroscopy, polarimetry, photoacoustic sensing, and optical coherence-based techniques, offer the possibility of remote, label-free, or reagent-free glucose detection. Fluorescence- and colorimetric-based sensors can provide rapid visual or optical responses through glucose-responsive chemical or enzymatic reactions [17], but they may face limitations in quantitative precision, long-term stability, photobleaching, and integration into practical wearable platforms. Raman spectroscopy provides molecular fingerprint information, while surface-enhanced Raman spectroscopy can significantly amplify weak Raman signals using plasmonic nanostructures such as gold or silver nanoparticles, nanopillars, and nanostructured films [18,19,20,21,22]. Despite these advantages, optical glucose sensing remains technically challenging because glucose has weak intrinsic optical signatures and measurements are often affected by tissue scattering, water absorption, motion artifacts, temperature, skin pigmentation, optical path-length variation, and interference from other biomolecules. Beyond the choice of transduction mechanism, the sampling matrix is an equally important determinant of sensor performance. This consideration has shifted attention from blood toward more accessible biological fluids that may support less burdensome monitoring.

Accordingly, tears, saliva, sweat, and interstitial fluid have attracted substantial interest as alternative matrices for glucose monitoring. The pursuit of painless glucose monitoring has also stimulated intense interest in alternative biofluids. Sweat, saliva, tears, and interstitial fluid can be accessed with lower discomfort than blood and are compatible with wearable formats such as epidermal patches, smart contact lenses, mouthguard sensors, microneedle arrays, and reverse-iontophoresis devices. These systems aim to improve user comfort, increase testing frequency, and enable continuous or semi-continuous monitoring outside clinical settings. However, alternative biofluids introduce additional complexity. Glucose concentrations in these fluids are often lower than in blood, may exhibit delayed correlation with blood glucose, and can be strongly influenced by local physiology, evaporation, contamination, sampling rate, pH, temperature, and mechanical deformation. Therefore, the translation of alternative-fluid sensors requires not only sensitive transduction mechanisms but also reliable sampling, calibration, biofluid handling, and validation against clinical reference standards.

Wearable glucose monitoring is increasingly becoming an interdisciplinary field at the convergence of biosensing, microelectronics, soft materials, data science, and connected health care. Next-generation platforms are expected to move beyond simple analyte detection toward integrated systems that combine sensing, signal processing, wireless communication, energy management, user feedback, and clinical decision support. Flexible and stretchable electronics can improve mechanical compatibility with skin and reduce motion artifacts. Microneedle technologies can access interstitial fluid with minimal pain while maintaining a closer correlation with blood glucose than many surface biofluids. Artificial intelligence and machine-learning algorithms may improve calibration, compensate for physiological variability, predict glucose trends, and integrate glucose data with contextual information such as diet, activity, sleep, heart rate, and medication use. These developments are creating new opportunities for personalized diabetes management, but they also raise important questions about reliability, data security, regulatory approval, long-term biocompatibility, affordability, and health equity access.

To maintain consistency across the diverse technologies discussed in this review, glucose-monitoring platforms are considered using a multi-axis classification framework rather than assigning each technology to a single mutually exclusive category. The first axis describes the degree of invasiveness, including invasive, minimally invasive, and non-invasive approaches. The second axis identifies the primary sampling matrix or physiological site, such as capillary blood, interstitial fluid, sweat, saliva, tears, urine, breath condensate, or intact tissue. The third axis describes the dominant transduction mechanism, including electrochemical, optical, spectroscopic, fluorescence, photoacoustic, microwave/radiofrequency, or field-effect-based sensing. A fourth, complementary descriptor is used when appropriate to identify system-level features such as wearable integration, wireless communication, artificial-intelligence-assisted analysis, or closed-loop therapeutic integration. Hybrid platforms are therefore not forced into a single category; instead, they are classified according to their primary physiological interface and dominant sensing mechanism, while their secondary sensing or computational components are explicitly identified. For example, a non-invasive microwave sensor combined with machine learning remains classified primarily as a non-invasive microwave platform, with AI/ML identified as a computational layer, whereas a microneedle-based electrochemical sensor is classified as minimally invasive, interstitial-fluid-based, and electrochemical. This framework allows technologies that combine multiple sensing or computational components to be compared without obscuring their underlying physiological sampling and transduction mechanisms.

**Figure 1:**
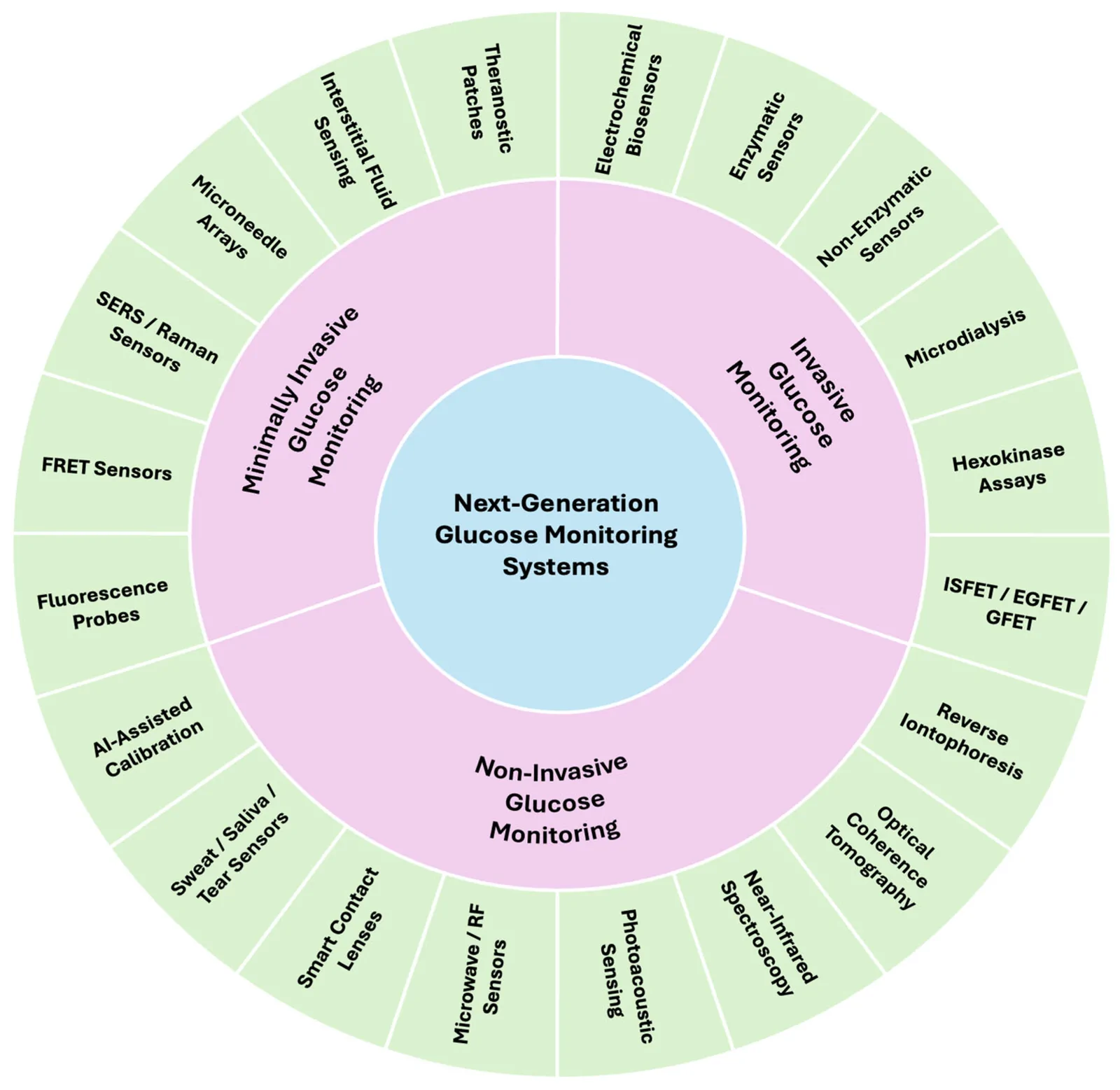
Taxonomy of glucose-monitoring technologies categorized by invasiveness and sensing modality.

Figure 1 presents the taxonomy of glucose-monitoring technologies categorized by invasiveness and sensing modality. Given the rapid expansion of this field, a comprehensive and critical review of glucose monitoring technologies is timely and necessary. This review examines the evolution of glucose sensing from traditional diagnostic methods to modern wearable and continuous sensing platforms. The author discusses invasive, minimally invasive, and non-invasive glucose monitoring strategies, with particular attention to electrochemical and optical transduction mechanisms, sensor materials, device architectures, sampling approaches, and performance-limiting factors. The author further highlights recent advances in continuous glucose monitoring, microneedle-based devices, flexible and wearable sensors, nanomaterial-enabled platforms, and emerging non-invasive technologies. Finally, the author identifies the major scientific, engineering, clinical, and translational challenges that must be addressed to realize the next generation of accurate, painless, affordable, and patient-centered glucose monitoring systems. By connecting historical progress with emerging innovations, this review aims to provide a clear perspective on how glucose monitoring technologies are evolving toward more intelligent, accessible, and integrated solutions for diabetes care.

From laboratory-based diagnostic tests and sporadic finger-prick glucometers, glucose monitoring has advanced to wearable biosensors, continuous glucose monitoring, and newly developed intelligent non-invasive systems. While electrochemical glucometers provide quick capillary blood testing, traditional diagnostics offer precise but sporadic readings. Wearable, continuous sensors provide trend-based glycemic data and extend monitoring to interstitial fluid.

**Figure 2:**
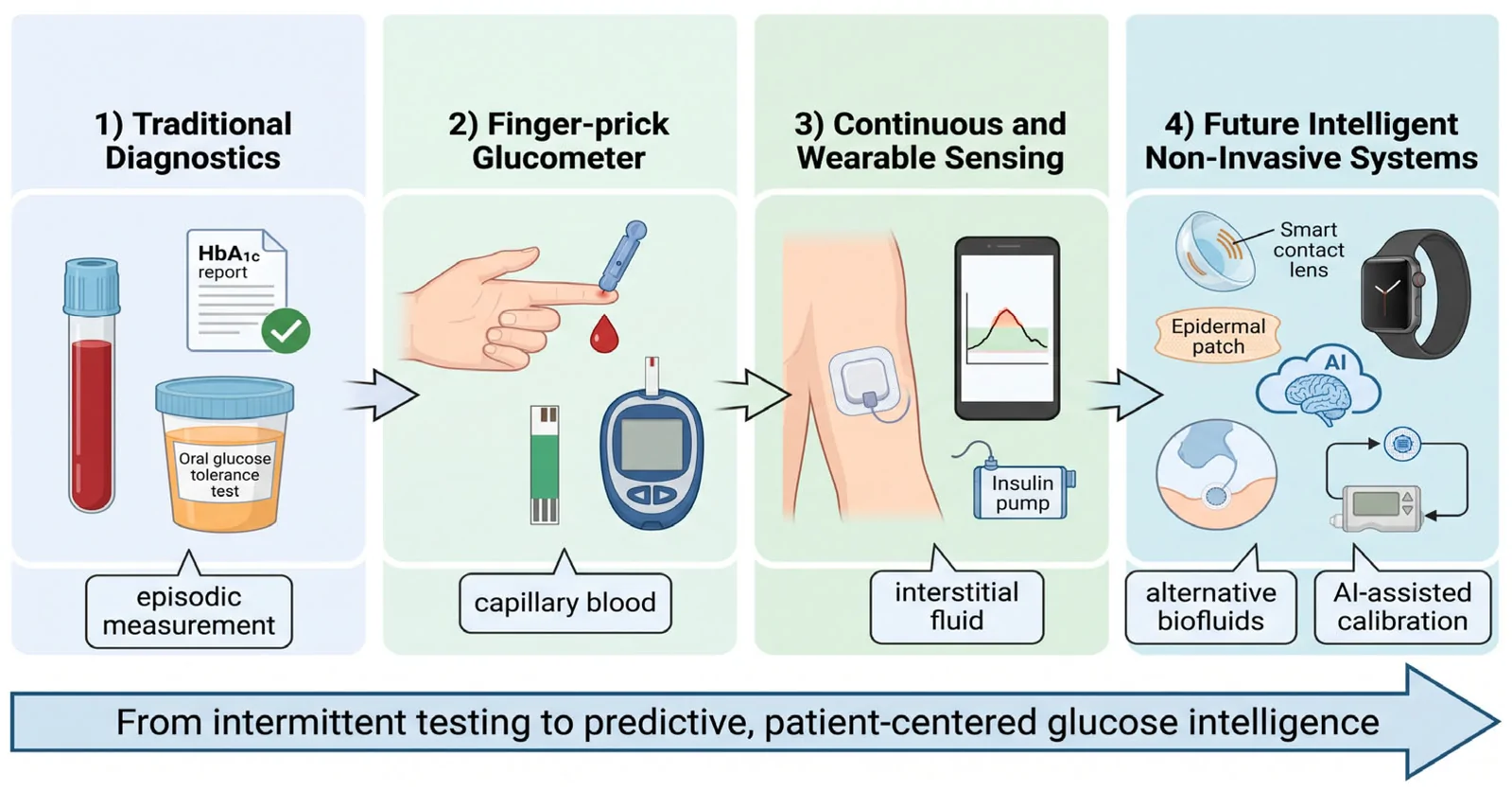
Evolutionary roadmap of glucose-monitoring technologies.

To provide predictive and patient-centered diabetes treatment, future systems are anticipated to incorporate alternative biofluids, smart contact lenses, epidermal patches, wireless monitoring, artificial intelligence-assisted calibration, and closed-loop insulin administration. Figure 2 displays an evolutionary roadmap of glucose-monitoring technologies.

## 2. Literature Search Strategy and Review Methodology

A narrative literature search identified representative studies describing the development, analytical performance, clinical translation, and technological challenges of glucose-monitoring systems. The author identified the literature using major scientific databases, including PubMed/MEDLINE, Scopus, Web of Science, IEEE Xplore, ScienceDirect, and Google Scholar. The search strategy combined terms related to glucose monitoring, biosensors, continuous glucose monitoring, wearable sensors, minimally invasive sensing, non-invasive glucose monitoring, electrochemical sensing, optical sensing, Raman spectroscopy, surface-enhanced Raman spectroscopy, near-infrared spectroscopy, optical coherence tomography, photoacoustic sensing, microwave and radiofrequency sensing, microneedles, alternative biofluids, smart contact lenses, artificial intelligence, and machine learning. The author used Boolean combinations of these terms to broaden or refine the search based on the technology under consideration.

The author prioritized peer-reviewed studies reporting experimental sensor development, analytical characterization, in vivo or clinical evaluation, device integration, calibration approaches, and translational performance. The author emphasized recent literature to reflect the rapid development of wearable, continuous, minimally invasive, non-invasive, and AI-assisted glucose-monitoring technologies, while retaining selected earlier studies that represented foundational technologies, landmark demonstrations, established clinical methods, or historically important developments. The author excluded studies unrelated to glucose sensing, those lacking sufficient methodological information, or those focused primarily on applications outside glucose monitoring. Because this article is a narrative and critical review rather than a systematic review or meta-analysis, the literature was synthesized thematically by invasiveness, sampling matrix, transduction mechanism, device architecture, analytical performance, and translational maturity. Particular attention was given to the distinction between analytical glucose detection and clinically meaningful glucose estimation, as well as to calibration, physiological variability, sensor stability, usability, and clinical validation. Table 1 shows the secondary/hybrid transduction strategy.

## 3. Invasive Glucose Monitoring: From Enzymatic Electrodes to Implantable Solid-State Biosensors

Invasive glucose monitoring encompasses methods that require blood collection or the transcutaneous insertion or subdermal implantation of a sensing component. These systems include finger-prick glucometers, implanted or transcutaneously inserted electrochemical sensors, and microdialysis-assisted devices. Most continuous invasive platforms measure glucose in interstitial fluid rather than directly in the blood, enabling temporally resolved assessment of postprandial excursions, nocturnal hypoglycemia, and rapid glycemic changes that may otherwise go undetected by intermittent capillary testing. By providing continuous or near-continuous measurements, these systems support treatment adjustment, trend analysis, and closed-loop insulin delivery. This section examines their sampling principles, transduction mechanisms, analytical performance, and clinical limitations.

Since it enables direct, high-frequency, and clinically actionable monitoring of glucose dynamics via subdermally implanted or transcutaneously inserted sensing devices, invasive glucose sensing remains a fundamental pillar of modern diabetes therapy. Unlike traditional blood glucose self-monitoring, which depends on sporadic capillary blood sampling, invasive and implantable platforms mainly measure glucose concentrations in interstitial fluid. This allows for a temporally resolved depiction of glycemic excursions, such as postprandial spikes, nocturnal hypoglycemia, and fast glucose fluctuations that might otherwise go unnoticed [23]. These technologies have increased the clinical relevance of glucose monitoring beyond episodic diagnosis to dynamic disease management, tailored treatment adjustment, and closed-loop insulin administration schemes by providing continuous or almost continuous biochemical observation. With a focus on electrochemical transduction, enzyme-mediated and enzyme-free detection mechanisms, microdialysis-assisted sampling, hexokinase-based photometric quantification, and ion-selective field-effect transistor architectures, this section critically examines advanced invasive glucose-monitoring modalities. Within this broad category, electrochemical sensors represent the most established platform because they combine rapid transduction with compact instrumentation and scalable manufacturing.

### 3.1. Electrochemical Glucose Biosensors: Enzymatic Recognition, Non-Enzymatic Catalysis, and Nanostructured Electrodes

Given their inherent compatibility with miniaturization, low-power operation, fast signal generation, high analytical sensitivity, and scalable manufacturing, electrochemical glucose biosensors are the most technologically advanced and commercially dominant class of glucose-monitoring systems. A working electrode, a reference electrode, and a counter electrode make up the three-electrode architecture used by most commercial glucometers [24]. In this arrangement, the working electrode converts glucose-dependent redox reactions into an electrical response, serving as the electroactive sensing interface. The reference electrode often uses silver/silver chloride (Ag/AgCl) or saturated calomel electrode (SCE) systems to maintain a steady and well-defined electrochemical potential. By facilitating charge balance and current passage through the electrolyte between the electrocatalytic medium and the working electrode, the counter electrode completes the electrochemical circuit. For the construction of counter-electrodes, highly conductive and chemically inert materials including graphite, glassy carbon, gold (Au), and platinum (Pt) are commonly utilized. The working electrode is the principal determinant of electrochemical sensor performance. Its surface morphology, catalytic activity, and electron-transfer kinetics influence glucose sensitivity and response time. Its functionalization chemistry, antifouling properties, and interfacial stability further determine selectivity, reproducibility, and operational lifetime [24]. After defining the electrochemical architecture, the next design consideration is the mechanism by which glucose is recognized and converted into an electrical signal. On this basis, electrochemical glucose sensors are divided principally into enzymatic and non-enzymatic platforms.

Enzymatic and non-enzymatic platforms are the two main categories of electrochemical glucose biosensors. Because glucose oxidase (GOD) offers remarkable molecular recognition toward glucose, high catalytic specificity, and robust analytical sensitivity when immobilized at the electrode interface, enzymatic glucose biosensors predominate in clinical and commercial applications. Enzymatic sensors currently predominate in clinical and commercial applications because immobilized glucose oxidase provides high molecular specificity and analytical sensitivity [25]. The GOD immobilization strategy is a critical biosensor-design parameter. It determines enzyme loading, molecular orientation, conformational stability, and substrate accessibility. It also affects electron-transfer efficiency, enzyme leaching, and long-term storage stability. The flavoprotein glucose oxidase uses molecular oxygen as the physiological electron acceptor to catalyze the oxidation of β-D-glucose to D-glucono-δ-lactone and hydrogen peroxide (H_2_O_2_) [25,26]. This reaction progresses mechanistically through linked oxidative and reductive half-reactions [26]. The flavin adenine dinucleotide cofactor inside GOD is reduced from FAD to FADH_2_ during the reductive half-reaction, while GOD oxidizes β-D-glucose to D-glucono-δ-lactone, which then undergoes non-enzymatic hydrolysis to gluconic acid. Molecular oxygen reoxidizes FADH_2_ during the oxidative half-reaction, producing H_2_O_2_ as a detectable electroactive byproduct. To modify the local peroxide content and affect the electrochemical readout, catalase (CAT) can further break down H_2_O_2_ into water and oxygen. Witteveen et al. identified lactonase (EC 3.1.1.17) from Aspergillus niger as the enzyme that catalyzes the hydrolysis of glucono-δ-lactone to gluconic acid [27].

Enzymatic glucose sensors are inherently limited by the physicochemical susceptibility of biological recognition elements, notwithstanding their shown usefulness. Temperature, humidity, pH, oxygen tension, chemical interferents, and long-term storage conditions can all significantly impact enzymatic activity. Thick enzyme layers may restrict glucose and oxygen transport to the catalytic sites. Poorly conductive immobilization matrices can also impede electron transfer between the flavin redox center and the electrode. These effects may reduce sensitivity and slow the sensor response [24]. Due to these limitations, much research has focused on non-enzymatic glucose sensing, which uses catalytic electrode materials to directly mediate glucose oxidation without the need for enzyme activity. Potential benefits of such systems include decreased biological degradation, enhanced resistance to enzyme denaturation, thermal stability, ease of manufacture, and operational longevity. These stability and electron-transfer limitations have motivated the development of enzyme-free sensing interfaces. Building on the electrochemical principles established by enzymatic sensors, non-enzymatic platforms replace biological recognition elements with catalytic electrode surfaces that oxidize glucose directly. The overall electrochemical oxidation process of glucose on metal surfaces was reported by Ernst et al. [28]. Because the hemiacetal hydroxyl group is more acidic than the alcoholic hydroxyl group, with pKa values of around 12.3 and 16, respectively, the hydrogen atom linked to the C1 carbon is preferentially activated for both α- and β-glucose. Thus, the primary product of electrochemical oxidation of α- and β-glucose is glucono-δ-lactone, which hydrolyzes to gluconic acid with a half-life of about 10 min at pH 7.5. On the other hand, gluconic acid may be directly produced by electrochemically oxidizing γ-glucose. Gluconic acid is the thermodynamically stable end product of glucose oxidation via a two-electron pathway, regardless of whether glucono-δ-lactone takes part as an intermediary [28]. The clinical success of electrochemical glucose sensing is most evident in conventional finger-prick glucometers, which translate these reaction mechanisms into rapid point-of-care measurements.

Conventional blood glucose monitoring uses a fingertip puncture to obtain a small capillary blood sample, which an enzyme-based glucometer subsequently analyzes [29,30]. A standard fingertip glucose meter combines a small signal-collecting, amplification, and processing module with a disposable or reusable glucose-monitoring electrode. GOD immobilized at the electrode surface is often the basis for the electrochemical sensing method. A small droplet of blood is obtained using a lancet during the measurement process and placed onto the sensor strip. The sample’s glucose is oxidized by enzymes, producing electrons or electroactive reaction products that change the current at the electrode interface. The signal collection system records this current, which is then transformed into a quantifiable glucose concentration using device-specific signal processing and calibration methods [29]. Finger-prick electrochemical monitoring is still often used in clinical, ambulatory, and home-care settings because of its quick reading, mobility, affordability, and simplicity of use. It allows customized therapy modification, assessment of pharmacological effectiveness, and risk reduction related to undiagnosed hypoglycemic or hyperglycemic episodes. Despite their widespread adoption, conventional enzyme-based strips remain constrained by enzyme degradation, interferent sensitivity, and limited opportunities for continuous operation. These shortcomings have redirected materials research toward enzyme-free and nanostructured electrodes.

Recent enzyme-free platforms therefore focus on engineering the working electrode with nanostructured catalytic materials. The environmental dependency of enzymatic activity, which includes susceptibility to temperature, humidity, pH change, oxygen availability, and electroactive interferents, limits the efficacy of enzyme-based glucose sensors despite their widespread clinical acceptance [24]. Enzyme-free electrochemical platforms have therefore received considerable attention, especially through advanced engineering of the working electrode using nanostructured catalytic materials [31], such as noble and transition metals [32], metal oxides [33], and carbon-based nanomaterials [34,35]. Nanomaterials are attractive for non-enzymatic glucose sensing because they provide high surface-to-volume ratios and abundant catalytic sites. Their tunable electronic structures can promote glucose adsorption, accelerate oxidation, and improve charge transfer. High electrical conductivity further supports rapid electrochemical signal generation. Their high electrical and thermal conductivity further supports the development of reliable, compact, and high-performing non-enzymatic sensors. For instance, Cherevko et al. [31] described a non-enzymatic electrochemical glucose sensor based on a gold nanowire array electrode. In this technology, glucose detection is controlled by partial and direct oxidation of glucose at the surface of the gold nanowire array. The sensor obtained a sensitivity of 41.9 μA mM^−1^ cm^−2^ and a detection limit of 30 μM [31]. Similarly, Zhai et al. used Enokitake mushroom-shaped Au nanowire films to create a highly stretchable electrochemical electrode that allowed glucose detection with a linear range of 0–800 μM under 30% strain and a sensitivity of 4.55 μA mM^−1^ cm^−2^ [36].

Metallic nanostructures offer high catalytic activity but may involve expensive materials and limited control over pore architecture. MOFs provide a complementary strategy through tunable coordination chemistry, high internal surface area, and structurally defined porosity. In addition to noble-metal nanostructures, low-cost, highly adjustable metal–organic framework (MOF)-based porous materials have become attractive options for electrochemical glucose detection. MOFs are useful for glucose sensing, energy storage, and electrocatalysis because of their large interior surface areas, well-defined porosity, adaptable coordination settings, high densities of catalytic sites, and opportunities for rational structural design [37]. Li et al. loaded a Co-MOF onto nickel foam to create a functional electrode. The resultant sensor showed an ultralow glucose detection limit of 1.3 nM and an extraordinarily high sensitivity of 10,886 μA cm^−2^ mM^−1^ [10]. Using a different approach, Imran et al. hybridized carbon nitride with several metals, such as ruthenium (Ru) and niobium (Nb), to create enzyme-free electrochemical glucose sensors [38,39]. These hybrid devices showed improved anti-interference behavior along with adequate stability, repeatability, and reusability, demonstrating the usefulness of heterostructure engineering for enhancing non-enzymatic glucose-monitoring performance.

Whereas metal- and MOF-based electrodes primarily improve catalytic activity and surface area, conductive polymers add mechanical flexibility and chemical processability. These properties are particularly valuable for wearable and conformable glucose sensors. Conductive polymers have emerged as useful glucose-sensing materials. They offer tunable conductivity, redox activity, chemical processability, and mechanical flexibility. Their compatibility with biological interfaces also supports their integration into wearable and implantable devices. Conductive polymers can achieve highly conductive states by chemical or electrochemical doping, and they usually have conjugated π-electron systems along their main chains. Polypyrrole (PPy), polyaniline (PANI), poly(phenylene vinylene) (PPV), and poly(3,4-ethylenedioxythiophene) (PEDOT) are examples of conductive polymers utilized in glucose-monitoring platforms [40]. By coupling the catalytic activity of copper oxide with the conductive network of polyaniline, Liu et al. created a glucose electrocatalyst [41]. It depicts the surface morphology of hollow PANI nanofibers. The resultant electrode showed a low detection limit of 0.45 μM and a wide detection range of 0.001–19.899 mM [41].

Electrode engineering improves transduction, but continuous monitoring also requires reproducible delivery of glucose from tissue to the sensing interface. Microdialysis addresses this sampling challenge by transporting interstitial glucose across a semipermeable membrane to an external or integrated detector. To improve real-time glucose tracking while preserving regulated subcutaneous tissue sampling, microdialysis-assisted sensing has also been implemented. A tiny hollow dialysis fiber is inserted into the subcutaneous compartment and continually supplied with isotonic fluid in microdialysis devices. After diffusing through the semi-permeable barrier and entering the perfusate, glucose from the surrounding interstitial fluid is moved to the outside for electrochemical measurement. This method allows continuous analyte extraction while providing regulated contact between the tissue and the sensor, which may reduce direct biofouling of the transducer. Recently, Najmi et al. developed an integrated, implantable microfluidic technology that uses microdialysis to measure glucose levels in the human body without the need for enzymes [42]. A 60 μm-tall microdialysis channel was combined with a non-enzymatic glucose sensor made of Pt–Ni nanoparticles and multiwalled carbon nanotubes on a screen-printed carbon electrode (Pt–Ni NPs–MWCNTs/SPE). This microfluidic sensor’s claimed detection limit of 21 μM was significantly higher than that of several of the previously mentioned non-enzymatic systems [42]. Zhao et al. created a highly stretchable electrochemical glucose sensor based on gold fibers for sweat glucose measurement in response to the increased interest in wearable and non-invasive glucose monitoring. The sensor maintained strain-insensitive and repeatable electrochemical performance throughout a strain range of 0–200% with a sensitivity of 13.9 μA mM^−1^ cm^−2^ by combining the intrinsic stretchability of conductive gold fibers with an extrinsic helical topology [43]. Although electrochemical systems dominate decentralized and continuous monitoring, laboratory glucose quantification still relies on highly specific reference assays. The hexokinase method provides an important benchmark against which emerging sensor technologies can be evaluated.

### 3.2. Hexokinase-Coupled Photometric Assays for Reference-Grade Glucose Quantification

The hexokinase method is a highly precise enzymatic measurement technique based on sequentially linked reactions, sometimes known as a photometric or spectrophotometric glucose test. In the initial stage, hexokinase phosphorylates glucose in the presence of magnesium ions and adenosine triphosphate (ATP), producing glucose-6-phosphate (G6P) and adenosine diphosphate (ADP). In the second stage, glucose-6-phosphate dehydrogenase oxidizes G6P in the presence of nicotinamide adenine dinucleotide (NAD), producing reduced nicotinamide adenine dinucleotide (NADH) and 6-phosphogluconate. The glucose level may be determined by measuring NADH absorbance at 340 nm using spectrophotometric detection since NADH production is stoichiometrically proportionate to glucose content [23,44]. The technique’s excellent specificity and direct relationship between optical absorbance and glucose concentration under carefully regulated reaction conditions make it a widely recognized reference-quality biochemical test.

Hexokinase activity can offer mechanistic understanding of cellular glucose use and metabolic control in addition to analytical glucose measurement. Hexokinase I and II were recently employed to measure metabolic rates linked to different subcellular distributions [45]. In Chinese hamster ovary cells, the effects of HKI and HKII on glucose consumption were examined. The findings indicated that HKI activity, which is mostly linked to mitochondria, improves glucose metabolism. In contrast, decreased glucose metabolism was linked to increased HKII activity, which is at least partially localized in the cytosol [45]. According to the results, cells may use variable hexokinase isoform localization as an adaptive mechanism to control glucose flow, maintain energy balance, and lessen their vulnerability to metabolic stress or damage. Therefore, hexokinase-based methods are useful for monitoring metabolic activity and cellular bioenergetic adaptability, as well as for quantifying glucose. Hexokinase assays provide high biochemical specificity but require reagents and optical instrumentation. Solid-state field-effect transistors offer a complementary route to compact and rapid glucose detection by converting interfacial biochemical changes directly into electronic signals.

### 3.3. Field-Effect Transistor Biosensors for Glucose Detection: ISFET, EGFET, and Graphene-Based Platforms

Invasive ion-selective field-effect transistor (ISFET)-based glucose sensors offer an alternative solid-state sensing architecture for monitoring glucose levels and can be inserted into or implanted beneath the skin to measure interstitial fluid glucose. ISFET glucose sensors usually convert chemical or metabolic interactions at the gate interface into changes in channel conductance, threshold voltage, drain-source current, or surface potential. The idea is connected to hydrogen peroxide electrolysis or local chemical changes produced as byproducts of glucose oxidation in several enzymatic ISFET glucose sensors. A field-effect transducer and a glucose-responsive sensor element make up a standard ISFET. The sensing film functions as the ISFET gate. Its material composition and surface functionalization influence molecular recognition. The dielectric properties, charge distribution, and interfacial chemistry determine the magnitude and stability of the electrical response [46].

An ISFET-based glucose sensor created by cross-linking a combination of glucose oxidase and bovine serum albumin in saturated glutaraldehyde vapor onto the transducer’s sensitive area was first shown by Vjacheslav Volotovsky et al. [47]. This method demonstrated the viability of combining field-effect-based glucose transduction with enzyme immobilization chemistry. More recently, Zhao et al. used dextran-capped silver nanoparticles (Dex-AgNPs) as sensitive nanoprobes on the ISFET gate to create a low-power, portable, real-time glucose-monitoring device [46]. In this setup, concanavalin A (Con A) was captured on the ISFET/Dex-AgNPs platform by using the carbohydrate-binding affinity between Con A and dextran. By competitively removing Con A from the ISFET/Dex-AgNPs/Con A complex through glucose–dextran competition, glucose was then indirectly detected [46]. This competitive-affinity design demonstrates how gate interfaces functionalized with nanomaterials may be designed to transform molecular recognition events into quantifiable field-effect signals.

Given graphene’s high carrier mobility, atomic-scale thickness, enormous specific surface area, superior electrical conductivity, and great sensitivity to interfacial charge perturbations, graphene field-effect transistor platforms have also been investigated. For instance, a graphene FET enzymatic biosensor encased in silk fibroin was created to detect glucose [34]. As glucose was oxidized by glucose oxidase immobilized inside the silk fibroin coating on the graphene FET surface, the device monitored differential drain-source current and Dirac point changes in the graphene transistor to determine glucose levels. The sensor showed a detection limit of 0.1 mM and a linear detection range of 0.1–10 mM [34]. Koike et al. fabricated the biosensing element of a TiO_2_/Ti extended-gate field-effect transistor (EGFET) by immobilizing a glucose oxidase-containing silk fibroin membrane on a TiO_2_/Ti surface to improve sensitivity further. The detection limit of this TiO_2_/Ti EGFET was 0.001 mg/mL [32].

Another intriguing method for amplifying glucose-dependent surface-potential changes through high-area semiconductor interfaces is through nanostructured oxide-based EGFETs. ZnO nanoarray-based EGFETs for glucose and pH monitoring were created by Junjie et al. [48]. Their sensor responded quickly, taking around 6–7 s [48]. The ZnO nanoarray’s high specific surface area and surface reactivity allowed the EGFET sensor to achieve a glucose sensitivity of −0.395 mV/μM throughout a 20–100 μM concentration range. These EGFET glucose biosensors were suitable for low-glucose biofluids like saliva and tears, where glucose levels are far lower than those usually seen in blood. They demonstrated high linearity and could detect glucose at concentrations as low as 20 μM.

Huang et al. created a polymer-functionalized graphene field-effect transistor (P-GFET) portable biosensing platform for glucose monitoring in order to improve mobility, reusability, and useful device integration. Acrylamide, 3-acrylamidophenylboronic acid (AAPBA), and N, N-dimethylaminopropyl acrylamide were used to create the functional polymer [49]. When glucose was present, the P-GFET showed current modulation and Dirac point changes due to the formation of covalent bonds between glucose molecules and AAPBA groups in the polymer layer on the graphene surface. For monitoring glucose in human urine, the P-GFET demonstrated a high sensitivity of 822 μA cm^−2^ mM^−1^ and a detection limit of 1.9 μM. With a detection range of 0.04–10 mM, the sensor was highly reusable and continued to function well after 20 cycles. These advancements show how promising ISFET, EGFET, and GFET designs are for portable, multipurpose, low-power, high-sensitivity glucose monitoring. To lower detection limits, reduce drift, increase gate stability, eliminate nonspecific adsorption, improve repeatability, and ensure dependable performance in complex physiological matrices, further tuning is still necessary.

Invasive glucose-monitoring techniques still face numerous obstacles despite their significant clinical and technological benefits, such as the need for calibration, discomfort during insertion, inflammatory reactions, foreign-body encapsulation, biofouling, tissue damage, sensor drift, a short operational lifetime, and variability at the implantation site. These biological and engineering limitations have accelerated the development of minimally invasive and non-invasive glucose-monitoring systems that aim to maintain analytical accuracy while enhancing user comfort, long-term wearability, and patient adherence.

**Figure 3:**
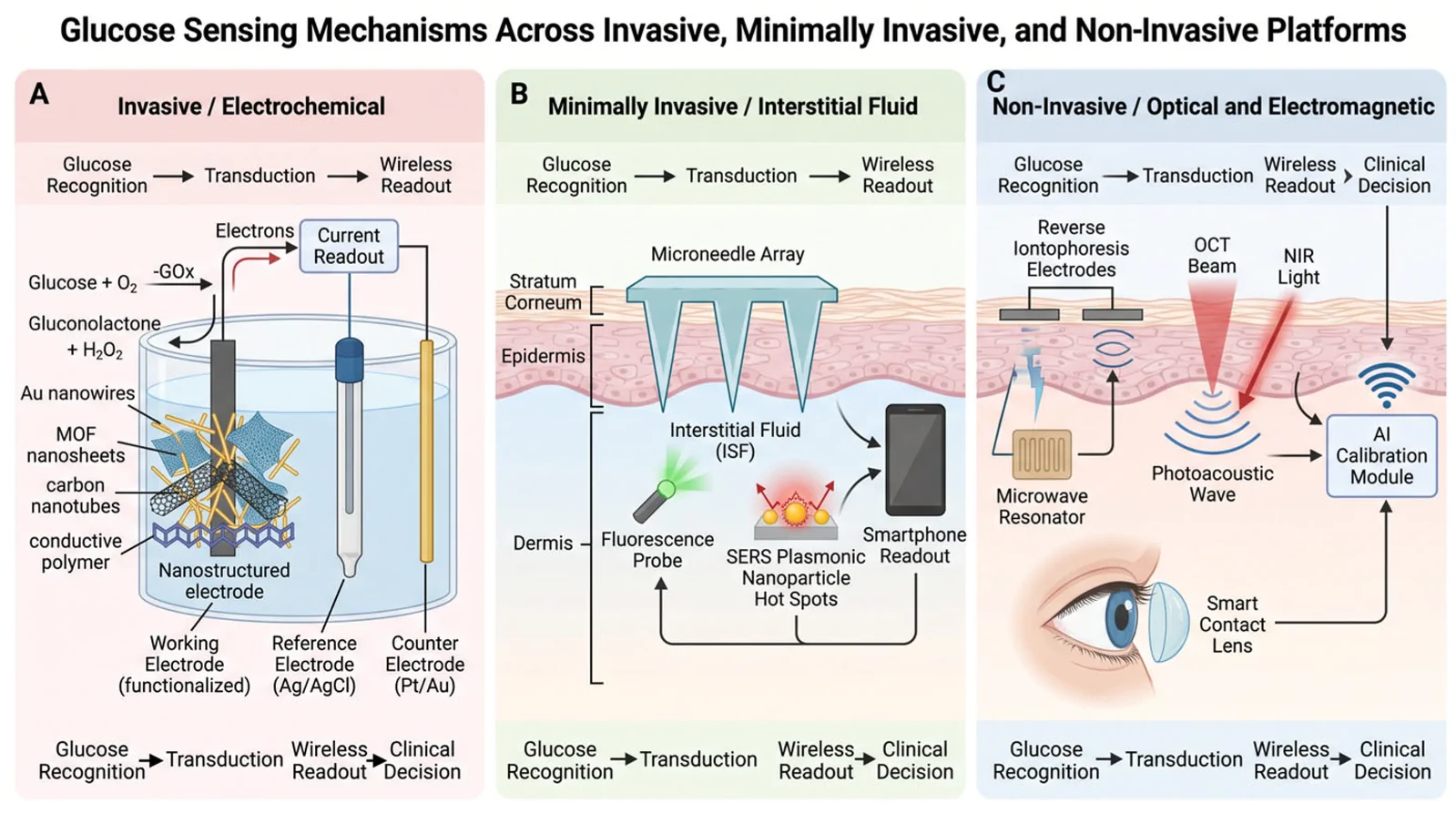
Sensing mechanisms across invasive, minimally invasive, and non-invasive platforms.

Three electrode combinations are frequently used in invasive electrochemical platforms to convert glucose oxidase-mediated or non-enzymatic glucose oxidation into current or potential data. Electrochemical, fluorescence, or surface-enhanced Raman spectroscopy readouts can be integrated into minimally invasive devices that use microneedle arrays to access interstitial fluid. Reverse iontophoresis, optical coherence tomography, near-infrared spectroscopy, photoacoustic sensors, microwave resonators, and smart contact-lens platforms are non-invasive methods that examine glucose-related signals through intact skin or alternative biofluids. For clinically significant glucose estimation across all modalities, glucose identification, signal transduction, wireless readout, and algorithmic calibration are essential. Figure 3 exhibits sensing mechanisms across invasive, minimally invasive, and non-invasive platforms. Electrochemical, microdialysis, and transistor-based systems provide sensitive glucose measurements, but their dependence on blood collection, sensor insertion, or implantation limits user comfort and prolonged acceptance. These constraints have accelerated the development of minimally invasive platforms that retain access to interstitial fluid while reducing penetration depth and tissue injury.

## 4. Minimally Invasive Glucose Monitoring: Optical Readouts and Microneedle-Mediated Interstitial-Fluid Sensing

Minimally invasive glucose monitoring includes technologies that cross the skin or another epithelial barrier with limited penetration and substantially less tissue disruption than conventional needles or implanted probes. Representative approaches include microneedle-mediated interstitial fluid sensing and shallowly implanted fluorescence or SERS-based probes. Although these platforms may reduce pain, bleeding, and infection risk, they should not be described as non-invasive because they still breach a biological barrier. Their principal advantage is the ability to access interstitial fluid or establish a localized sensing interface while minimizing injury to blood vessels, nerves, and surrounding tissue.

A crucial translational link between entirely non-invasive monitoring systems and traditional blood-based tests is provided by minimally invasive glucose-monitoring devices. Their main goal is to provide clinically accurate glucose data while significantly reducing discomfort, tissue damage, infection risk, sample burden, and behavioral fatigue associated with repeated finger-prick measurements. Minimally invasive systems use shallow skin penetration or localized access to interstitial fluid. Some platforms employ subcutaneous optical or biochemical interfaces. These approaches reduce disruption of vascularized tissue and nociceptive structures compared with conventional needles or deeply implanted probes. Compared to many external biofluids, these systems can thus offer a more pleasant and continuous monitoring experience while preserving a tighter physiological link to blood glucose.

Fluorescence-based sensing techniques [16,17,50], surface-enhanced Raman spectroscopy (SERS)-based sensing [18,19,51], and microneedle-based sensors [52] are a few minimally invasive glucose-monitoring systems that have garnered substantial scientific interest. The transduction physics, analyte-recognition chemistry, biointerface requirements, and integration potential of these systems vary. Microneedle arrays offer controlled microscale access to interstitial fluid while avoiding major blood vessels and nerve endings; SERS-based systems use plasmonically amplified vibrational fingerprints for molecularly specific glucose detection; and fluorescence-based platforms translate glucose–probe interactions into changes in optical intensity, wavelength, lifetime, or ratiometric ratios. Taken as a whole, these modalities represent a rapidly developing class of minimally disruptive glucose-monitoring systems that have the potential to provide real-time, continuous, and customized diabetes care. The sensing mechanisms, materials engineering, device topologies, analytical performance, and translational problems of exemplary minimally invasive techniques are highlighted in this section.

### 4.1. Fluorescence-Based Glucose Sensors: Boronic Acid Probes, FRET Systems, and Hydrogel Platforms

As fluorescence-based glucose sensing combines molecular sensitivity with relatively inexpensive instrumentation, rapid readout, and compatibility with miniaturized or implantable forms, it has become a highly useful optical technique for bioanalysis and biomedical monitoring. High analytical sensitivity, good selectivity, visual detectability, spatiotemporal traceability, and the possibility of minimally invasive or non-invasive monitoring are among the benefits of these methods. The working idea is based on either glucose-responsive recognition systems or glucose-dependent modification of fluorescence probes. Glucose–probe interactions can alter fluorescence intensity, emission wavelength, or fluorescence lifetime. They may also change energy-transfer efficiency, anisotropy, or a ratiometric signal [50]. Fluorescence-based techniques are appealing for decentralized glucose monitoring because fluorescence readout can be achieved with small, affordable optical components, such as visible-light-emitting diodes, ultraviolet light sources, photodiodes, charge-coupled device detectors, and smartphone-compatible imaging modules [53,54,55].

The integration of optical signal transduction with molecular identification is a key design need in fluorescence-based glucose sensing. Receptors serve as enzymatic labels, glucose-recognition elements, affinity mediators, or fluorescence-modulating elements in most systems. These receptors are designed to either directly bind glucose or participate in glucose-dependent processes that result in observable spectral shifts, ratiometric changes, fluorescence amplification, or quenching. For instance, carbon quantum dots (CQDs) and o-diaminobenzene (ODB) were used to create a recently developed colorimetric and ratiometric fluorescence sensor [55]. This method enabled quantitative detection of glucose using ratiometric fluorescence and colorimetry and semi-quantitative detection by monitoring color changes at 365 nm upon exposure to sunlight or UV radiation [55]. These dual-mode optical techniques are especially useful because they combine straightforward visual readout with instrument-based quantification, thereby increasing accessibility and potential point-of-care applications.

The analytical performance of a fluorescence sensor depends largely on how molecular recognition is coupled to optical transduction. Several receptor classes have therefore been investigated to provide reversible or reaction-based glucose recognition. Boronic acid derivatives [56], glucose-binding proteins like concanavalin A (Con A) [57,58], enzymes [59], quantum dots [35,60], carbon nanotubes [17,61], and other nanomaterial-based optical probes are just a few of the many receptor chemistries and fluorescent transducers that have been investigated for glucose detection. Because of its tetrameric shape, which offers four carbohydrate-binding sites and permits robust, reversible interactions with glucose-containing ligands, Con A continues to be particularly significant for affinity-based glucose sensing. A glucose sensor based on fluorescence resonance energy transfer (FRET) was recently developed by Tang et al. using quantum dots and plasmonic gold nanoparticles connected by Con A [62]. Nanoscale separation controls the fluorescence response of the quantum dots and gold nanoparticles in this arrangement, which function as donor–acceptor components. Due to its greater affinity, Con A preferentially binds to glucose upon its addition, causing the quantum dots and AuNPs to separate and changing the fluorescence intensity [62]. This device illustrates how FRET modulation can convert competitive affinity recognition into a distance-dependent optical readout. FRET systems provide sensitive distance-dependent readouts, but continuous in vivo operation additionally requires chemical and photophysical stability. Hydrogel encapsulation offers one strategy for protecting glucose-responsive probes within physiological environments.

Enhancing fluorescence sensors’ operational stability in physiological settings continues to be a top research focus. A recent work used boronic acid-regulated fluorescence intensity as the transduction mechanism to provide a hydrogel-based glucose sensor with improved in vivo durability [63]. In order to shield arylboronic acid recognition motifs from hydrogen peroxide-mediated oxidative degradation in vitro, the sensor included two antioxidant enzymes, superoxide dismutase and catalase. For almost 28 days, the hydrogel glucose sensor’s boronic acid-based fluorescence response was maintained in vivo by this antioxidant defense. Anthracene fluorescence is muted in the absence of glucose [63]; however, fluorescence intensity rises when glucose binds to arylboronic acid groups. This approach emphasizes the significance of hydrogel encapsulation, control of reactive oxygen species, and molecular protection in sustaining long-term optical sensor performance following implantation.

Beyond direct fluorescence modulation, glucose can also trigger controlled probe release. Enzyme-gated mesoporous materials exploit this mechanism to amplify the optical response through glucose-induced chemical changes. Aznar et al. created a gated mesoporous silica nanodevice, another advanced nanochemical design, in which cyclodextrin-modified glucose oxidase (CD-GOx) and methylbenzimidazole moieties at the pore openings interacted supramolecularly to retain ruthenium bipyridine complexes inside silica mesopores [64]. Under baseline conditions, this design stopped the fluorescent probes from being released too soon. When glucose was added, glucose oxidase produced gluconic acid, which disrupted the gatekeeping interaction and led to fluorescence recovery. This system demonstrates how mesoporous nanocarriers, pH-responsive gating, enzymatic catalysis, and fluorescence release may all be combined into a single glucose-responsive platform. Because they enable signal amplification via controlled probe release rather than relying solely on direct probe–glucose binding, such gated nanodevices are highly attractive.

There have been reports of other fluorescence biosensor architectures that use carbon dots as donors and graphene oxide as an acceptor [65], europium sulfosuccinimidyl dextran as the donor and Alexa Fluor 647 sulfosuccinimidyl as the acceptor [66], and quantum dots as donors and dextran-bound malachite green as an acceptor [67]. The variety of FRET, ratiometric, and nanomaterial-assisted fluorescence techniques accessible for glucose detection is demonstrated by these devices. Sensitivity, dynamic range, and biological compatibility can be optimized with significant flexibility by tuning donor–acceptor separation, spectral overlap, quantum yield, and surface chemistry. Czarnik et al. [68] described a modified fluorescent chemosensor combined with a sophisticated algorithm for real-time blood glucose monitoring [68].

The practical appeal of fluorescence sensing also stems from its cost, accessibility, and compatibility with subcutaneous implantation without requiring significant surgical procedures. Numerous studies have investigated contact-lens-based glucose sensors for easy tear-fluid monitoring due to these characteristics [69,70]. By adding boronic acid groups to its surface, Seo et al. created a smart contact lens [70]. A poly(tannic acid) functional coating was applied to the lens surface in order to promote boronic acid conjugation. The lens was able to bind glucose present in biological fluids such as tears thanks to this change. The collected glucose was released by submerging the lens in an enzymatic solution and then measured using a colorimetric assay [70]. Contact-lens platforms integrate glucose sensing into an established wearable format. Their translation nevertheless requires a reliable relationship between tear and blood glucose. Ocular safety, tear-production variability, optical transparency, mechanical comfort, and long-term lens stability must also be established.

Fluorescence-based glucose monitoring still has a number of enduring drawbacks despite significant advancements. While photobleaching can degrade long-term optical stability, biological autofluorescence from endogenous fluorophores can reduce measurement specificity and compromise the signal-to-background ratio [71]. Photon scattering, tissue absorption, optical path-length variations, probe encapsulation, inflammatory responses, and subject-specific skin characteristics, such as pigmentation levels, all have an impact on in vivo fluorescence readout [72]. These problems can lower quantitative dependability and cause calibration drift. Near-infrared and second-near-infrared fluorophores, fluorescence lifetime imaging, ratiometric self-referencing probes, anti-fouling hydrogel matrices, photostable nanomaterials, implantable optoelectronics, and algorithmic correction for tissue-dependent optical variability are likely to be key components of future developments. Fluorescence platforms offer sensitive and potentially low-cost readout, but their performance can be limited by photobleaching, autofluorescence, and tissue-dependent attenuation. Raman spectroscopy provides a complementary optical strategy by identifying glucose through molecule-specific vibrational signatures.

### 4.2. Raman and Surface-Enhanced Raman Glucose Sensing: Plasmonic Enhancement, Molecular Fingerprinting, and In Vivo Potential

By examining inelastic light scattering associated with specific chemical bonds, Raman spectroscopy, a highly selective vibrational spectroscopic method, enables label-free molecular identification. It is particularly appealing for identifying biomolecules in complex biological matrices due to its ability to produce chemically precise spectral signatures. Previously, a variety of biological samples and physiological parameters, such as glucose concentration, pH, and redox potentials, have been analyzed using Raman-based technologies [12,13,14]. Conventional Raman detection is limited by the small scattering cross-section of glucose and its low physiological concentration. Glucose bands may also overlap with biological background signals. In addition, glucose adsorbs poorly onto many unmodified plasmonic surfaces. Due to these restrictions, surface-enhanced Raman spectroscopy platforms have been developed extensively. These platforms use plasmonic nanostructures, such as silver or gold nanopillars, nanoparticles, and nanostructured films, to amplify weak Raman signals by factors that generally range from 10^6^ to 10^8^ [20,21,22].

To overcome these intrinsic signal limitations, SERS concentrates the electromagnetic field near plasmonic nanostructures and amplifies Raman scattering from molecules located within nanoscale hot spots. Localized surface plasmon resonances that generate strong electromagnetic fields near nanostructured metallic surfaces underlie SERS’s remarkable sensitivity. Raman scattering is significantly increased for molecules in these near-field locations, especially at interparticle junctions or nanoscale “hot spots.” However, the key design problem for glucose sensing is not only signal augmentation but also molecule localization: glucose may not stay in the plasmonic hot-spot area long enough for repeatable measurement and lacks a high inherent affinity for many bare noble-metal surfaces. To concentrate glucose near the metallic surface while maintaining reversible binding and reducing nonspecific adsorption, SERS glucose sensors often incorporate self-assembled monolayers, boronic acid derivatives, thiol linkers, or other molecular capture layers.

Signal enhancement alone is insufficient because glucose has limited affinity for unmodified noble-metal surfaces. Functional capture layers are therefore required to localize glucose within the effective near-field region. Stuart et al. created implantable nanospheres using self-assembled monolayers and a silver coating. By acting as chemically selective interfacial layers on the silver screen, the SAMs improved analyte signals and made in vivo SERS-based applications possible [73]. Silver nanoparticles applied to microneedle surfaces enhance Raman signals in microneedle-integrated SERS systems, and a 1-decanethiol coating acts as a glucose-capturing layer to concentrate glucose molecules from interstitial fluid close to the plasmonic substrate [74]. Because it addresses both sampling and detection issues by combining minimally invasive interstitial fluid access with plasmonically enhanced molecular readout, this design is very appealing.

Yang et al. created a SERS glucose sensor using gold-coated zinc oxide nanowires functionalized with 4-mercaptophenylboronic acid [75]. The final substrate was used to test glucose ex vivo [75]. The viability of dynamic glucose monitoring in biomedical settings was supported by the reversible binding of 4-mercaptophenylboronic acid to glucose, enabling continuous monitoring of ambient glucose concentrations [75]. Because it forms reversible cyclic boronate esters with cis-diol-containing compounds such as glucose, boronic acid chemistry is particularly useful in this context. This creates a regenerable affinity interface for repeated or continuous sensing.

SERS sensitivity, stability, and biological compatibility are all significantly impacted by material choice. Silver nanoparticles can offer better sensitivity because they typically produce a greater SERS enhancement than gold nanoparticles [76,77]. Silver nanoparticles exhibit greater surface plasmon effects than gold nanoparticles under similar circumstances, according to several studies [76,78]. Olga et al. coated polystyrene nanospheres with silver and modified their surfaces with mercaptohexanol and decanethiol to create a SERS-based glucose-monitoring substrate. A glucose detection limit of 0.55 mM was attained by this technique [51]. However, glucose may be positioned outside the highest near-field enhancement region by large linker molecules, thereby increasing unpredictability and decreasing signal intensity. When the molecular recognition layer is excessively thick or spatially diverse, it is challenging to get consistently accurate measurements due to the distance-dependent degradation of the SERS electromagnetic field [75,79].

A SERS-based glucose nanosensor for in vitro measurements was recently created by Dey et al. With outstanding compatibility, the nanosensor probe demonstrated a linear response over the glucose concentration range of 1–10 mM and reached a detection limit of 0.1 mM. To track ongoing metabolic activity in various tumor microenvironments, the nanosensor was incorporated into tumor spheroids. Over five days, intratumoral glucose was assessed. Compared to monocultured fibroblast spheroids, the glucose gradient produced by co-cultured colon cancer spheroids was almost twice as high [18]. This finding sheds light on metabolic heterogeneity in three-dimensional tumor models, as cells in the core experience significant glucose deprivation, whereas cells in the periphery receive greater direct nutritional availability from the surrounding medium.

While Raman and SERS-based methods offer significant opportunities for ongoing glucose monitoring, there are still several technical and translational obstacles to overcome. High-end equipment, reliable optical alignment, controlled acquisition settings, precise calibration procedures, and skilled operators are often required for Raman systems. Batch-to-batch variability, variable hot-spot distributions, fouling, signal drift, restricted regeneration, and matrix-dependent spectrum interference are all potential problems for SERS substrates. Furthermore, implanted or minimally invasive SERS devices must meet strict criteria for biocompatibility, mechanical durability, optical accessibility, and long-term stability. These restrictions may be gradually overcome with further developments in repeatable nanofabrication, plasmonic substrate standardization, internal-reference techniques, spectrum deconvolution, downsized Raman equipment, and machine-learning-assisted calibration. All things considered, Raman-based glucose sensors have great potential for next-generation minimally invasive and continuous monitoring applications, especially where spatially resolved metabolic tracking with chemical specificity is needed. Raman and SERS platforms provide high molecular specificity, but implantable optical probes still require stable access to the target biofluid. Microneedle arrays address this interface problem by creating microscale pathways to interstitial fluid with limited tissue disruption.

Another challenge for SERS-based glucose monitoring is managing spectral noise and substrate-dependent signal variability. Because SERS enhancement depends strongly on nanoscale electromagnetic hot spots, variations in nanoparticle size, spacing, morphology, surface chemistry, and analyte localization can produce substantial spatial and batch-to-batch differences in signal intensity. Recent work has therefore explored substrate-level approaches for improving spectral uniformity and signal-to-noise ratio (SNR). For example, hybrid SERS substrates incorporating two-dimensional materials such as graphene, MoS_2_, and WSe_2_ with gold nanoparticles have been investigated as noise-management strategies; Au nanoparticle/graphene substrates were reported to substantially reduce spectral noise and improve SNR relative to conventional Au nanoparticle substrates [80]. At the optical-interface level, roughening the terminal surface of fiber-optic SERS probes has also been shown to reduce background contributions and improve SNR, providing a practical strategy for remote SERS measurements [81]. Signal-processing approaches provide a complementary route to noise suppression. Wiener-estimation-based spectral reconstruction has been demonstrated to recover Raman spectra acquired at low SNR and has been compared favorably with conventional Savitzky–Golay, finite-impulse-response, wavelet, and factor-analysis approaches [82]. For glucose monitoring, these approaches could be combined with internal spectral standards, baseline correction, multivariate analysis, reproducible substrate fabrication, and machine-learning-based spectral interpretation. However, computational denoising should be carefully validated to ensure that noise suppression does not remove glucose-associated spectral features or artificially improve apparent prediction performance. Thus, effective SERS noise management should be considered as a system-level problem encompassing substrate engineering, optical design, acquisition protocols, and computational signal processing.

### 4.3. Microneedle Array Sensors: Low-Trauma Access to Interstitial Fluid for Continuous Monitoring

Microneedle arrays provide direct access to interstitial fluid with less pain and bleeding than conventional needles. They may also reduce tissue trauma and foreign-body burden relative to deeply implanted probes. These advantages make microneedle-based glucose monitoring a promising minimally invasive strategy. Microneedles are usually shorter than around 1 mm and have micrometer-scale diameters [83]. These microscale structures bypass deeper blood vessels and nerve endings while penetrating the stratum corneum and extending into the viable epidermis or superficial dermis when assembled in arrays [83]. Because of their anatomical selectivity, microneedles may avoid the skin’s primary barrier function without causing the pain and tissue damage that come with conventional hypodermic insertion.

The design and production of microneedle platforms have advanced significantly over the last 20 years, resulting in a variety of structural formats, including solid, hollow, coated, porous, hydrogel-forming, and dissolvable microneedles. Because they may directly sample or examine interstitial fluid, whose glucose content closely tracks systemic glycemic dynamics, hollow and sensing-integrated microneedles are especially appealing for glucose monitoring. Additionally, insertion depth, mechanical strength, fluidic transport, analyte diffusion, and sensor–tissue contact may all be controlled by engineering the microneedle’s design. For instance, Ribet et al. integrated an ultra-miniaturized electrochemical sensing probe into a single hollow microneedle to create a continuous glucose-monitoring system [52]. The apparatus demonstrated a linear response up to 14 mM glucose and a sensitivity of 1.5 nA/mM. Reduced pain and great accuracy during real-time monitoring were shown in in vivo human testing [52]. This study demonstrates that it is possible to achieve minimally invasive interstitial fluid glucose monitoring by directly embedding electrochemical sensing in a hollow microneedle.

A wearable continuous glucose monitoring device based on microneedles, operated via a smartphone for real-time glucose testing, was introduced by Jian and colleagues [84]. The platform measures subcutaneous glucose levels and accesses interstitial fluid using hollow microneedles. Stable electrochemical performance and high sensitivity are achieved with flexible sensing electrodes fabricated via screen printing and laser cutting. With a relative standard deviation of 4.8%, the glucose sensor, which uses a sandwich-type enzyme immobilization technique for in vitro detection, demonstrated high measurement accuracy and repeatability [84]. The use of wireless electronics, flexible electrodes, microneedle sampling, and smartphone integration is part of a broader trend toward patient-centered, digitally connected glucose monitoring devices.

By creating a fully integrated wearable microneedle array that can wirelessly, continuously, and in real time monitor two metabolites in interstitial fluid during normal daily activities, Wang and colleagues substantially advanced the field [85]. The system consists of a reusable electrical device that can be broken down into nine parts and a disposable sensor. Practicality and cost-effectiveness can be increased by replacing the low-cost disposable part when the sensor wears out or runs out while keeping the reusable electronics. The disposable sensor’s microneedle electrodes pierce the skin to reach the interstitial fluid, where they electrochemically detect analytes such as glucose and lactate. After that, the wearer’s gadget receives the recorded signals wirelessly for processing and analysis [85].

Sun et al. combined regulated insulin release with glucose sensing to create a multifunctional microneedle patch for diabetes management, expanding microneedle platforms from passive monitoring to therapeutic intervention [86]. Both glucose-responsive insulin administration and visual glucose measurement are made possible by the patch. The tips of the microneedles are coated with glucose oxidase and mineralized insulin particles after being treated with 3-aminophenylboronic acid and crosslinked with chondroitin sulfate. Glucose oxidase catalyzes the conversion of glucose to hydrogen peroxide under hyperglycemic conditions. The calcium-containing mineral layer dissolves due to the hydrogen peroxide produced, thereby promoting prolonged insulin release. Concurrently, the horseradish peroxidase layer at the patch’s base changes color due to hydrogen peroxide, allowing for visual glucose detection [86]. An essential step toward closed-loop or self-regulated transdermal diabetic treatment is this integrated sensing-and-release device.

Microneedle-based glucose sensors are becoming more affordable, safe, and useful thanks to materials engineering. Yuen et al. demonstrated a silver-coated agarose microneedle design in a skin-mimicking phantom. This method improved biocompatibility and cost-effectiveness while achieving excellent sensitivity, on par with that of stainless steel microneedles [87]. Ju et al. also developed a novel biosensor for in situ, in vivo measurements using a polymer-based methyl methacrylate microneedle array. These investigations show that it is possible to reconcile mechanical penetration, electrochemical sensitivity, biological compatibility, manufacturing scalability, and patient comfort by customizing microneedle composition and design.

When combined, microneedle technologies offer a potent path toward next-generation continuous glucose monitoring by combining a wearable, minimally invasive interface with the physiological influence of interstitial fluid sensing. They are at the vanguard of customized diabetes-management technologies due to their compatibility with wireless communication, multiplexed metabolite detection, flexible electronics, smartphone-based control, and glucose-responsive medication administration. However, before being widely used in clinical settings, several issues need to be resolved, including consistent insertion depth, long-term skin compatibility, mechanical durability, enzymatic stability, calibration drift, biofouling, inflammatory response, motion artifacts, sweat interference, sterilization, regulatory validation, and scalable manufacturing. The development of microneedle glucose sensors from lab prototypes to dependable, clinically approved, and widely available monitoring systems is anticipated to be accelerated by ongoing advancements in microneedle materials, microfabrication, soft bioelectronics, antifouling coatings, enzyme immobilization, and integrated closed-loop algorithms. Microneedle systems substantially reduce tissue trauma, yet they still penetrate the skin and therefore remain minimally invasive. The pursuit of barrier-free monitoring has consequently shifted attention toward technologies that interrogate intact tissue or externally accessible biofluids.

## 5. Non-Invasive Glucose Monitoring: Transdermal Extraction, Tissue Optics, and Electromagnetic Sensing

Non-invasive glucose monitoring refers to the acquisition of glucose-related information without penetrating the skin or another epithelial barrier. These technologies interrogate intact tissue, extract analytes across intact skin, or analyze externally accessible biofluids using optical, electromagnetic, electrochemical, or transdermal mechanisms. Representative approaches include reverse iontophoresis performed across intact skin, optical coherence tomography, infrared spectroscopy, photoacoustic sensing, microwave and radiofrequency platforms, and tear-based smart contact lenses. In contrast, systems that incorporate microneedles or implanted components are classified in this review as minimally invasive or invasive, even when they are painless or integrated with a nominally non-invasive measurement modality.

The development of genuinely non-invasive monitoring reflects the demand for continuous glycemic information without repeated skin penetration. If clinically validated, these platforms could reduce discomfort and infection risk while improving adherence and access to longitudinal glucose data. One of the most ambitious goals in diabetes technology during the last decades of the 20th century was the development of non-invasive glucose monitoring. An ideal glucose-monitoring system would provide reliable, continuous, and real-time glycemic information without penetrating the skin. Eliminating repeated finger-prick sampling or implanted sensors could reduce pain, infection risk, tissue injury, and user burden. To provide painless and convenient metabolic monitoring, non-invasive glucose sensors aim to detect glucose-related physiological signatures via intact skin, sweat, tear fluid, urine, optical scattering, electromagnetic response, or transdermal extraction. By enhancing patient adherence, facilitating early detection of hypo- and hyperglycemia, supporting closed-loop insulin delivery, reducing the burden of frequent sampling, and expanding glucose surveillance for broader preventive and wellness applications, such platforms, if clinically validated, could significantly transform diabetes care.

Non-invasive glucose sensing remains one of the most technically challenging areas in biomedical engineering, despite its great promise. Glucose generally produces a weak and often indirect signal in biological tissue. Water, proteins, lipids, electrolytes, and pigments can dominate the measured response. Tissue heterogeneity, perfusion, motion, temperature, hydration, and interindividual anatomical differences introduce additional variability. As a result, low signal-to-background ratio, calibration instability, physiological latency, device–skin coupling inconsistency, and inadequate sensitivity at clinically significant glucose concentrations are common problems with non-invasive measures. However, this subject has gained fresh impetus due to persistent developments in soft electronics, optical instrumentation, nanophotonics, electrochemical interfaces, microwave engineering, computational modeling, and artificial intelligence [88]. Reverse iontophoresis, optical coherence tomography, infrared absorption spectroscopy, photoacoustic and microwave-based techniques, and smart wearable platforms are among the main non-invasive glucose-monitoring system techniques in this area.

### 5.1. Reverse Iontophoresis: Electro-Osmotic Extraction of Glucose Across Intact Skin

A low-intensity electrical current can be used to sample trace compounds from intact skin via reverse iontophoresis, a transdermal extraction method. Reverse iontophoresis removes endogenous molecules from the skin and interstitial compartment toward the electrode interface, whereas standard iontophoresis pushes charged therapeutic substances into the skin. This method uses electro-osmotic solvent flow to move neutral glucose molecules over the epidermal barrier for glucose monitoring. The basis for later advancements in this technology was laid in 1991, when Richard H. Guy’s team proposed using reverse iontophoresis for transdermal glucose extraction as a diagnostic technique for diabetes [89].

A modest electric current is sent across the skin between two electrodes during reverse iontophoresis. Under physiological conditions, glucose is electrically neutral; hence, electrophoretic migration is not the primary mode of transport. Rather, the charged skin microenvironment generates an electro-osmotic flow that transports glucose [90]. Because the skin has a net negative charge at physiological pH, Na^+^ ions are the predominant mobile counterions. Na+ migration causes convective solvent flow from the anode to the cathode when an electric field is applied. This solvent flux entrains neutral solutes such as glucose and transports them to the iontophoretic cathode, where they can be electrochemically measured [91]. Therefore, skin permeability, charge density, ionic strength, moisture, current density, electrode shape, and extraction kinetics all influence the analytical performance of reverse iontophoresis in addition to the glucose sensor itself.

As the first transdermal glucose sensor licensed by the US Food and Drug Administration, the Cygnus GlucoWatch reverse-iontophoresis sensor marked a significant historical milestone [91,92]. GlucoWatch showed adequate accuracy in clinical testing for several home blood glucose monitoring applications [93]. Its clinical usefulness, however, was limited, especially for detecting hypoglycemia. For glucose readings of ≤3.3 mmol/L, the device’s sensitivity was only 23%, and its false-alarm rate was 51% [94]. The device was discontinued in 2008 due to these constraints, as well as problems such as skin discomfort, sweat-induced artifacts, calibration requirements, delayed results, and fluctuating extraction efficiency. The GlucoWatch experience is still instructive, as it illustrated the viability of non-invasive electro-extraction as well as its translational challenge: analytical precision alone is not enough unless comfort, dependability, usability, and hypoglycemic performance are all clinically acceptable. Although GlucoWatch demonstrated the feasibility of transdermal glucose extraction, its clinical limitations highlighted the need for better skin interfaces, more sensitive detectors, and improved compensation algorithms. Advances in flexible electronics have enabled renewed investigation of this approach.

Reverse-iontophoresis-based monitoring has gained popularity again due to advances in flexible electronics, skin-conforming electrodes, microfabricated electrochemical interfaces, and wearable wireless systems. To increase sweat production and monitor analytes such as glucose with improved integration and sensitivity, a number of transdermal and miniaturized iontophoresis interfaces have been developed [95,96]. For instance, Emaminejad et al. developed an electrochemically enhanced iontophoresis interface that can simultaneously measure sweat analytes such as glucose, Na^+^, and Cl^−^ and stimulate localized sweat production [95]. On-site and real-time sweat analysis was made possible by the platform’s integration of sensing electrodes on the same substrate as the iontophoresis electrodes. The device is made up of a wireless flexible printed circuit board connected to an electrode array for both sweat induction and sensing, as seen in [95]. Crucially, the design reduces crosstalk and allows each module to work independently by physically and functionally separating sweat stimulation from analyte detection. The stated glucose sensitivity was just 2.1 nA/μM, despite the wearable platform’s significant demonstration of integrated non-invasive biochemical monitoring. Sweat-based iontophoretic systems preserve the skin barrier but often recover only low glucose concentrations. Hybrid platforms seek to increase analyte access by combining reverse iontophoresis with controlled microscale barrier disruption.

Cheng et al. developed a touch-activated glucose biosensor to monitor interstitial fluid glucose levels with increased sensitivity, thereby improving signal quality and extraction efficiency [96]. Three functional modules are integrated into this system: an electrochemical sensor unit for glucose detection, a reverse-iontophoresis unit for interstitial fluid extraction, and a microneedle array for painless stratum corneum disruption [96]. The sensing interface was created by immobilizing glucose oxidase in an agarose gel at the working electrode. Enzymatic electrochemical detection, electrically aided reverse-iontophoretic extraction, and shallow skin penetration make up the device’s sequential workflow [96]. With a sensitivity of 7.76 μA mM^−1^, the sensor significantly outperformed previous wearable sweat-based iontophoretic devices. Zhang et al. presented a novel biofuel cell-based sensing system in a separate study that combines flexible enzymatic biofuel cells with a power management patient circuit to detect glucose in urine and serve as an alert for diabetes with diabetes [97]. This idea emphasizes the potential for low-power or self-powered biochemical sensors that directly link energy collection and signal creation to glucose oxidation.

Despite these developments, reverse iontophoresis still has several issues that must be addressed before it can be used as a reliable glucose-monitoring method in clinical settings. First, compared with the quantity in the blood, the amount of glucose taken across intact or partially disrupted skin is usually much smaller. This calls for electrochemical sensors with extremely low detection limits, excellent selectivity, quick reaction times, and robust resistance to skin metabolites, sweat components, and electrochemical interferents. Second, consistent electrical connection and stable skin adhesion are necessary for reverse iontophoresis. Human skin is curved, deformable, viscoelastic, and spatially heterogeneous. Rigid devices may therefore lose conformal contact during movement. This can produce pressure gradients, delamination, motion artifacts, and local variations in current density, thereby altering extraction efficiency and sensor output. Soft, elastic, breathable, and skin-conforming materials that can sustain sustained electrochemical contact during movement, perspiration, and prolonged usage are therefore essential for next-generation devices.

Third, the concentration of glucose in sweat or extracted interstitial fluid differs from that in blood. Converting extracted analyte levels into clinically significant blood glucose values requires establishing a trustworthy link between these compartments [98]. Physiological lag, microcirculatory variability, local perfusion, skin hydration, temperature, sweat rate, tissue transport kinetics, and inter-subject variations, however, confound this mapping [99]. Future reverse-iontophoresis systems will thus need physiologically based calibration models that include adaptive data analytics, transdermal transport modeling, and sensor physics. Skin permeability, electro-osmotic flow, and sensor response are further influenced by environmental factors such as temperature and humidity, which emphasizes the necessity of integrated temperature/humidity sensing algorithmic correction. In the end, co-optimization of extraction efficiency, sensor sensitivity, mechanical conformance, biocompatibility, and computational calibration will be necessary for efficient reverse-iontophoresis glucose monitoring. Reverse iontophoresis relies on extracting glucose across the skin and is therefore sensitive to local transport and skin properties. OCT follows a fundamentally different strategy: it estimates glucose indirectly by measuring glucose-associated changes in tissue optical scattering.

### 5.2. Optical Coherence Tomography: Depth-Resolved Tissue Scattering for Glucose Estimation

Based on low-coherence interferometry, optical coherence tomography is a non-invasive, depth-resolved optical imaging method. Examining the interference between light reflected from a reference arm and light backscattered from tissue provides micrometer-scale structural information. Because near-infrared light provides a good compromise among tissue penetration, scattering contrast, small optical components, and biological safety, it is typically used in Optical Coherence Tomography (OCT) [100]. A typical OCT system comprises an interferometer and a photodetector or camera-based detection module. The light source is divided between sample and reference arms. Light backscattered from tissue recombines with light reflected from the reference mirror. The resulting interference signal encodes depth-dependent optical-path information. Glucose-related changes in tissue optical properties may alter the detected signal at depths of up to approximately 1.6 mm.

OCT glucose sensing is based on the theory that glucose modifies tissue scattering by altering the refractive-index matching between cellular or extracellular structures and the interstitial fluid. The interstitial fluid’s refractive index varies with increasing glucose content, altering the refractive-index mismatch between scatterers and the surrounding medium. The tissue scattering coefficient, which may be measured using OCT signal slope, attenuation, speckle statistics, or decorrelation characteristics, may be decreased or altered in some other way. The glucose-induced optical shift, however, is small and competes with much bigger fluctuations caused by motion, blood flow, temperature, mechanical pressure, tissue hydration, and structural heterogeneity.

OCT-based glucose detection was shown to be feasible in early in vivo investigations. Larin et al. found a correlation (R = 0.88) between OCT-derived signals and blood glucose levels in vivo; however, motion artifacts and skin temperature significantly affected the findings [101,102]. These findings demonstrated that OCT could detect glucose-related optical changes, but they also highlighted the need for rigorous control of physiological and mechanical confounders. GlucoLight created an OCT-based glucose monitor in the late 2000s [103]. Twelve patients with type 1 diabetes and fifteen with type 2 diabetes participated in a pilot clinical investigation in 2008 [104]. The technology produced a mean absolute relative difference of 11.5% after two hours of measuring per participant. Nevertheless, hypoglycemic-range readings were not included in the paper, and no further published research on the device has been documented. This lack of follow-up highlights a persistent problem with non-invasive glucose monitoring: promising short-term performance must be verified across large populations, prolonged wear times, hypoglycemic ranges, and practical working environments.

Su et al. introduced a three-dimensional correlation technique that exploits OCT volumetric imaging [105]. This approach examines three-dimensional OCT datasets to identify and exclude tissue regions with weak or non-correlating scattering behavior, rather than relying on one-dimensional or two-dimensional signal correlations. Compared with previous 1D and 2D correlation techniques, the method significantly improves estimates of blood glucose concentration (BGC) by eliminating low-information or confusing regions during data processing. To minimize motion artifacts and maintain steady contact pressure between the probe and the skin, the authors also developed an optical probe in the shape of a bracelet. The OCT system was combined with the wristband-style optical probe. 88.4% of data points fell within Zone A, indicating a high degree of clinical accuracy, according to a clinical evaluation using Clarke error grid analysis [105]. This study demonstrates that multidimensional signal processing and mechanical stabilization can significantly enhance OCT-based glucose estimation.

However, OCT-based glucose monitoring remains largely at the prototype stage. Optical system downsizing, component costs, motion sensitivity, long-term signal drift, probe–skin coupling variability, and anatomical variations between subjects are among the main obstacles [106]. Additionally, skin temperature, humidity, hydration, local perfusion, pressure-induced blanching, and variations in microstructural tissue all influence OCT signals [106]. Accurate glucose assessment requires complex feature extraction, motion correction, physiological compensation, and customized calibration because glucose-induced variations in scattering are minimal compared with these confounding factors. Higher-stability light sources, smaller integrated photonics, better optical probes, multimodal sensing, adaptive algorithms, and extensive clinical validation across a variety of skin types, glycemic ranges, and environmental circumstances will all be necessary for future advancements. OCT provides depth-resolved structural information but detects glucose indirectly through subtle scattering changes. Infrared spectroscopy instead targets wavelength-dependent molecular absorption, while photoacoustic and microwave systems exploit complementary acoustic and dielectric responses.

### 5.3. Infrared, Photoacoustic, Microwave, and Smart Contact-Lens Platforms for Non-Invasive Glucose Monitoring

Through measuring the wavelength-dependent absorption and scattering of infrared light, infrared absorption spectroscopy may identify molecular vibrational and rotational transitions. Because near-infrared spectroscopy provides deeper tissue penetration than mid-infrared light and is well-suited to wearable optical setups, fiber optics, small sources, and photodiodes, it has been studied most extensively for non-invasive glucose sensing. The normal NIR spectral range is between 780 and 2500 nm, or 14,000 and 4000 cm^−1^. The first overtone region at about 6500–5500 cm^−1^ and the combination band region at about 5000–4000 cm^−1^ are frequently used to measure glucose because they include absorption characteristics associated with the stretching and bending vibrations of C–H and O–H in glucose. Because NIR photons may permeate tissue sufficiently for transdermal measurements, these overtone and combination bands are nonetheless appealing despite being weak, wide, and partially overlapping with water and tissue absorptions.

Usually, transmittance or reflectance mode is used for NIR spectroscopy. The sample is exposed to polychromatic light in transmittance mode from a source such as an LED or tungsten-halogen lamp. A diffraction grating is used to split the transmitted light into its component wavelengths, which are subsequently examined by a computer and detector. The source and detector lenses are placed on the same side of the tissue in reflectance mode, and the reflected light is collected at a predetermined angle following spectral dispersion [103]. Diffuse reflectance spectroscopy is better suited for in vivo non-invasive glucose monitoring because it permits light to pass through skin and return to the detector without requiring optical access to the opposite side of the tissue. In contrast, transmission spectroscopy is frequently used for aqueous solutions and thin samples [103].

Numerous investigations in humans and animals have demonstrated quantifiable relationships between NIR signals and glucose content [107,108,109], sparking ongoing interest in NIR spectroscopy. Olesberg et al. showed how to use a fiber-optic probe to measure glucose in rats. The device achieved a standard error of prediction of 35.6 mg/dL (1.98 mM) and spanned a detection range of 90–630 mg/dL (5–35 mM) [107]. Additionally, clinical studies have been carried out. Maruo and associates developed a non-invasive NIR glucose sensor for patients with diabetes and compared the outcomes with those of healthy individuals. Seven patients with diabetes and five healthy individuals participated in the study. Over a glucose range of 50–500 mg/dL, the reported standard error of prediction was 27.2 mg/dL [109]. The comparison of reference and forecasted glucose readings, together with the Clarke error grid analysis [109].

Biological noise is a significant drawback of NIR glucose detection. Glucose absorption, water absorption, hemoglobin concentration, lipid content, collagen structure, melanin, epidermal thickness, dermal scattering, tissue temperature, pressure, moisture, and blood perfusion all contribute to the optical signal obtained from the skin. Information specific to glucose may be obscured by these variables, which can fluctuate more than glucose itself does. Han et al. developed a two-step system-improvement approach to reduce interindividual and measurement-condition variability. To lessen the effect of human variability, a specially designed multi-ring indium gallium arsenide detector with a high signal-to-noise ratio was employed. Second, to replicate the reliable measurement geometry, a fixation-and-targeting approach was devised. Volunteers with diabetes and those in good health underwent seventeen glucose tolerance tests. With an average correlation of 0.84 between the optical signal and reference glucose across 17 glucose tolerance tests, the results showed that blood glucose fluctuations could be detected at 1550 nm [108].

Researchers are increasingly using neural network-based algorithms and multi-band NIR data to improve prediction accuracy. While machine-learning algorithms may capture nonlinear correlations among optical characteristics, physiological confounders, and reference glucose levels, multi-band NIR offers a better spectral representation of tissue–glucose interactions. Garcia et al. monitored glucose responses in vitro in aqueous solution using an artificial neural network that included NIR and 37–39 GHz stimulation [110]. Nevertheless, the lack of individualized medical elements in many older models limited their ability to account for individual anatomical and physiological variations. NIR responses can be significantly disrupted, and prediction accuracy can decrease due to interference from bone, tissue thickness, fatty tissue, moisture, and other biological variables. Srichan and associates created a multi-photonic-band NIR sensor combined with shallow, dense neural networks and customized medical features to solve this problem [111]. Srichan and associates developed a multi-photonic-band NIR sensor combined with a shallow dense neural network and personalized medical features. Datasets from 401 blood samples were randomized for model development using ten-fold validation, while a separate cohort of 234 individuals not included in the model-training dataset was used for evaluation. The model achieved a classification accuracy of 97.8%, with 94.8% sensitivity, 96.0% precision, and 98.7% specificity for diabetes mellitus classification using a fasting blood glucose threshold of 126 mg/dL. These metrics describe classification performance rather than continuous glucose concentration-estimation accuracy. For non-invasive glucose estimation, the study additionally reported a detection range of 60–400 mg/dL and an error of approximately ±15% with a 95% confidence interval, using the standard hexokinase method as the reference. Accordingly, classification metrics and continuous glucose-estimation metrics should be interpreted separately when assessing the clinical performance of AI-assisted glucose-monitoring systems [111]. By using machine-learning methods for multiple-wavelength measurements for glucose monitoring, Shokrekhodaei et al. furthered this trend [112].

Despite these developments, NIR spectroscopy is still not used in any widely used commercial glucose-monitoring gadget. The main challenge is not just optical detection but also clinical precision that can be applied across diverse demographics and real-world settings. Skin pigmentation, sunburn, perspiration, tattoos, tissue hydration, and temperature can alter the measured spectrum. Probe pressure and anatomical measurement site introduce further variability. These factors limit the transferability of calibration models across individuals and settings. For accurate optical signal interpretation across patients and physiological conditions, customized calibration models remain necessary. Future NIR systems will likely require domain-generalizable machine learning, adaptive calibration, physiological covariate sensing, integrated multispectral or hyperspectral data collection, and rigorous prospective validation against reference glucose levels. The weak and overlapping absorption bands encountered in NIR spectroscopy have encouraged investigation of modalities that convert optical or dielectric interactions into alternative signal domains. Photoacoustic sensing transforms absorbed optical energy into acoustic waves, whereas microwave resonators detect glucose-associated changes in dielectric properties.

Several other non-invasive sensing techniques, such as photoacoustic spectroscopy [113] and microwave resonators [114], have been investigated alongside NIR spectroscopy, OCT, and reverse iontophoresis. Acoustic detection and optical excitation are combined in photoacoustic spectroscopy. This method creates localized thermoelastic expansion and ultrasonic pressure waves when laser light is absorbed by tissue chromophores or target molecules. These pressure changes, which rely on optical absorption, are detected by an ultrasonic transducer [113]. According to earlier in vivo research, photoacoustic techniques may detect glucose with higher sensitivity than traditional spectroscopic methods [113]. The poor photoacoustic response of water, which makes it easier to detect signals linked to glucose and hydrocarbons in aqueous biological settings, is partially responsible for this increased sensitivity.

Since glucose-induced changes in dielectric characteristics can modify the resonant frequency, amplitude, phase, or quality factor, microwave resonator-based sensing has also emerged as a promising method. Baghelani et al. developed a microwave resonator-based sensor for continuous monitoring of interstitial fluid glucose. The sensor uses a split-ring resonator tag without a substrate, which is electromagnetically coupled to a reader. Microwave resonator-based glucose sensors may be appropriate for incorporation into artificial pancreas systems, as the sensing element does not need direct power consumption [114]. Resonance-based glucose sensors in the microwave range have been the subject of several additional investigations. According to one study, a metal-insulator-semiconductor micro-resonator might serve as a trustworthy glucose indicator [115]. A very sensitive resonator-based microwave biosensor for real-time blood glucose detection was described by another [116]. Additionally, real-time continuous glucose monitoring has been assessed using radiofrequency-based biosensors [117].

Although microwave resonators support passive and flexible sensing, wearable implementation also requires mechanically compliant power, communication, and display components. Smart contact lenses illustrate how these functions can be integrated into a compact ocular platform. Building on these advancements, soft wearable and ocular platforms have been developed that include wireless power transmission, sensing, and real-time output into small, non-invasive devices. A soft smart contact lens with wireless power-transfer circuits, glucose sensors, and display pixels for real-time glucose viewing is a prime example [118]. The hybrid lens construction developed by Park and associates comprises elastic joints for stretchy parts such as antennas and interconnects, mechanically reinforced islands for electronic devices, and transparent, stress-tunable materials [118]. To maintain optical clarity, the hybrid materials were selected to reduce refractive index mismatch, resulting in 93% transparency and 1.6% haze. Ocular health depends on oxygen permeability, which is enhanced by the elastic areas. One-dimensional ultralong metal nanofibers were used to create stretchable transparent electrodes. An external transmitter within 9 mm of the antenna in this smart lens transmits radiofrequency signals to the antenna. The alternating-current RF signal is converted into direct-current power by a rectifier made of a diode and capacitor, which powers the glucose sensor and LED [118].

The glucose sensor’s resistance decreases when the tear glucose concentration exceeds a certain threshold. While the resistance of the antenna, rectifier, and other circuit elements is constant, this lowers the overall resistance of the parallel circuit that houses the LED and glucose sensor. Consequently, under constant voltage, the bias applied to the LED-sensor circuit varies, allowing the LED to turn on or off based on glucose concentration. A graphene-based sensor in this device detects tear glucose, and the embedded LED display wirelessly displays the data. Non-invasive glucose monitoring is made possible by the platform without the need for cumbersome external equipment. Safety and dependability were validated by in vivo rabbit tests. This smart contact lens architecture may enable broader applications in medication administration, augmented reality, and continuous health monitoring, in addition to glucose monitoring. Significant technological obstacles remain, including low signal-to-noise ratio, RF interference, tear-film variability, sensor fouling, power-transfer efficiency, ocular biocompatibility, and difficulty distinguishing glucose from other tear constituents. These difficulties highlight the need for enhanced system-level integration, selectivity, sensitivity, and stability.

As artificial intelligence and machine learning can identify weak glucose-dependent characteristics from noisy, multidimensional physiological inputs, they are increasingly seen as crucial enabling technologies for non-invasive glucose monitoring. As long as the training data are sufficiently vast, diverse, and clinically representative, data-driven models can account for subject variability, environmental changes, sensor drift, nonlinear tissue responses, and multimodal interference. A non-invasive, wrist-worn device that uses artificial intelligence to evaluate changes in microwave resonance was the subject of a pilot investigation by Qureshi et al. Bulk plasma glucose levels are measured by the device using a dial-resonating sensor, and the results are converted into glucose values. Using a random-sample technique applied to the entire dataset, the device predicted plasma glucose across four cohorts, with an average mean absolute relative difference of 10.3% [119]. This study demonstrates the increasing confluence of wearable technology, electromagnetic sensing, and AI-driven calibration. In the future, robust sensing physics, physiologically informed device design, multimodal data acquisition, and adaptive computational models that can maintain clinical accuracy under real-world conditions are likely to be the most successful non-invasive glucose-monitoring systems. Collectively, these platforms demonstrate substantial progress toward painless glucose monitoring. Their clinical value, however, depends on more than analytical detectability; it requires stable calibration, biological specificity, reproducibility, safety, usability, and validation under real-world conditions. These cross-cutting constraints are examined in the following section.

The table presents a comparative overview of major glucose-monitoring technologies by invasiveness, sampling matrix, and transduction strategy, as reported for invasive, minimally invasive, and non-invasive glucose monitoring technologies. It lists the main glucose-monitoring technologies by sensor matrix, transduction principle, benefits, drawbacks, and sample literature, providing an organized comparison across invasive, minimally invasive, and non-invasive platforms. Table 2 presents a comparative Overview of major glucose-monitoring technologies by invasiveness, Sampling Matrix, and Transduction Strategy.

## 6. Established Commercial Continuous Glucose Monitoring Systems

Established CGM systems provide an important clinical benchmark for evaluating emerging glucose-monitoring technologies. Current commercial platforms predominantly use subcutaneous sensors to measure interstitial-fluid glucose and provide near-continuous glucose estimates, trend information, alerts, and integration with smartphones, cloud-based platforms, and, in selected systems, automated insulin-delivery technologies. Their principal clinical advantage is not simply generating frequent measurements, but providing longitudinal glucose profiles that support assessment of glycemic variability, time in range, hypoglycemia, hyperglycemia, and treatment response. The extensive clinical use and regulatory evaluation of commercial CGM systems also provide a much more mature translational foundation than most experimental non-invasive platforms.

Nevertheless, established CGM systems should not be considered without limitations. Because their sensors measure interstitial rather than directly circulating blood glucose, physiological lag can become relevant during rapid glucose excursions. Sensor insertion, finite sensor lifetime, skin irritation, biofouling, signal drift, device failure, and dependence on calibration and signal-processing algorithms may affect user experience and measurement performance. Economic cost and unequal access can also limit sustained use in some populations. Importantly, these limitations do not diminish the clinical value of commercial CGM; rather, they establish the performance and usability benchmark that emerging minimally invasive and non-invasive systems must meet or exceed before they can reasonably replace established CGM or finger-prick testing. Consequently, experimental platforms should be evaluated using clinically meaningful measures of agreement, error, reliability, wearability, and patient benefit rather than analytical sensitivity alone. Table 3 presents representative commercial continuous glucose monitoring systems and key clinical characteristics.

## 7. Existing Limitations, Performance Bottlenecks, and Translational Challenges in Glucose-Monitoring Technologies

Through enabling more frequent glycemic assessments, better medication adjustment, and early detection of hypo- and hyperglycemic excursions, the development of glucose-monitoring devices has significantly changed diabetes diagnosis, monitoring, and therapeutic management. These developments have improved glucose management and reduced the risk of diabetes-related complications. Despite decades of progress, no existing platform satisfies all requirements for ideal glucose monitoring. Such a system would need reliable performance across clinically relevant glucose ranges and during rapid glycemic change. It would also require long-term stability, biocompatibility, affordability, minimal calibration, resistance to biofouling and environmental variation, and compatibility with continuous use in daily life.

**Figure 4:**
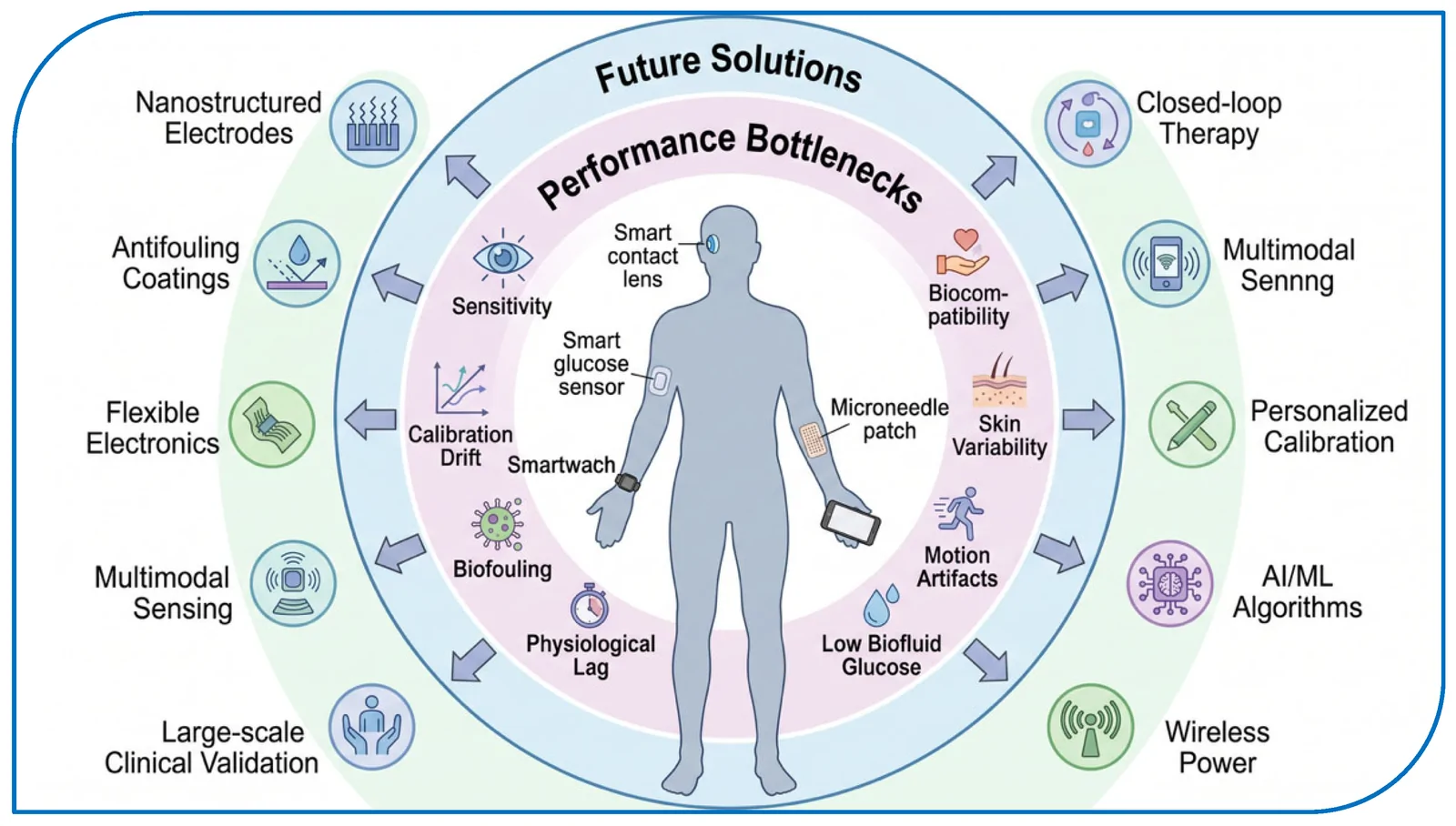
Translational challenges and future engineering priorities.

Inadequate sensitivity or selectivity, calibration drift, biofouling, physiological lag, motion artifacts, skin variability, low glucose concentrations in alternative biofluids, and long-term biocompatibility issues are among the enduring problems with current glucose-monitoring devices. Nanostructured electrodes, antifouling coatings, flexible electronics, multimodal sensing, wireless power transfer, patient-specific calibration, artificial intelligence and machine-learning algorithms, large-scale clinical validation, and closed-loop therapeutic integration are all necessary for future advancements. Developing precise, painless, continuous, and patient-centered glucose monitoring devices requires addressing these obstacles. Figure 4 shows translational challenges and future engineering priorities.

An important distinction should be made between detecting glucose within an alternative biofluid and accurately reconstructing blood glucose concentration from that biofluid. Detecting glucose in sweat, saliva, tears, urine, or other matrices shows that the analyte is measurable in the sampled fluid but does not, by itself, establish that the measured concentration can reliably serve as a surrogate for blood glucose. For clinical glucose monitoring, an alternative-biofluid platform must additionally demonstrate a reproducible and physiologically meaningful relationship between the measured biofluid signal and blood glucose across changing glycemic conditions. This relationship may be affected by transport delay, local secretion and dilution, fluid turnover, hydration, pH, temperature, contamination, sampling rate, tissue perfusion, and inter-individual physiological variability. Therefore, studies reporting alternative biofluid glucose detection should distinguish analytical detection performance from blood-glucose reconstruction performance and should report validation against an appropriate reference blood-glucose measurement. Demonstrating a low detection limit or strong correlation within a controlled sample matrix should not be interpreted as equivalent to clinically accurate continuous blood-glucose estimation.

Due to their high biochemical specificity and well-developed device infrastructure, enzyme-based glucose sensors remain the most clinically proven and commercially effective glucose-monitoring devices. However, because they rely on biological recognition elements, enzyme-based glucose sensors [64,131] have inherent limitations. Heat denaturation, humidity-induced deterioration, pH fluctuations, chemical instability, oxygen dependency, and interference from electroactive or redox-active substances in biological fluids can all affect enzymes such as glucose oxidase and glucose dehydrogenase. These elements may affect long-term operating stability, shorten shelf life, decrease sensitivity, and impair repeatability. Furthermore, the ongoing expense of replacing sensing components and disposable enzyme-functionalized strips remains a significant financial burden, especially for patients who require ongoing monitoring or frequent testing. Non-enzymatic sensors and other biofluid-based monitoring systems have been proposed as solutions to these issues; nonetheless, it remains challenging to simultaneously achieve high sensitivity, selectivity, repeatability, stability, and clinical reliability in complex physiological matrices [15].

Due to their portability, affordability, ease of use, and minimally disruptive, non-invasive operation, wearable sensors for point-of-care glucose monitoring in biofluids have attracted considerable attention [120]. This reduces patient discomfort and increases the potential for continuous monitoring. The shift from episodic glucose testing to decentralized, customized metabolic monitoring largely depends on these platforms. However, the biofluid sampled, the physiological relationship between that biofluid and blood glucose, the stability of the sensor–biofluid interface, and the device’s capacity to continue operating reliably in the face of motion, temperature fluctuations, perspiration, mechanical deformation, and prolonged wear all have a significant impact on wearable glucose sensor performance.

Since blood glucose measurement most accurately reflects systemic glycemic state, it continues to be the gold standard for both diagnosis and treatment. As a result, wearable blood glucose monitors have been the subject of much study. Many blood-based wearables, however, still need skin punctures, subcutaneous sensor insertions, or blood contact, making them invasive or semi-invasive. Pain, discomfort, tissue damage, infection risk, local inflammation, and decreased patient adherence are all associated with these needs, especially when measurements need to be taken frequently or continuously [132]. Furthermore, physiological lag, calibration drift, biofouling, foreign-body reaction, limited sensor lifespan, and unpredictability arising from implantation-site conditions can affect even sophisticated continuous glucose monitoring systems that detect interstitial fluid rather than blood.

Sweat, urine, tears, and saliva are examples of alternative biofluids that have been extensively studied as accessible, non-invasive matrices for glucose monitoring. Due to their non-invasive accessibility and compatibility with wearable or disposable sensing formats, sweat and urine are particularly appealing [95,97,133]. Significant promise has been shown by wearable sweat sensors based on gold fibers [95], biofuel-cell-based systems for urine glucose detection [97], and tear-based sensors incorporated into soft contact lenses [118]. For instance, to enable real-time visualization of glucose levels, smart contact lenses can incorporate wireless power-transfer circuits, glucose sensors, and display pixels. Stretchable electrodes made of ultralong metal nanofibers and translucent, stress-tunable materials are used in these lenses. Bulky external equipment is no longer necessary because glucose signals from tear fluid can be wirelessly transmitted to an LED-based display device. Sensor drift, low signal-to-noise ratio, interference from adjacent radiofrequency devices, difficulty distinguishing glucose from other biological components, and insufficient sensitivity and accuracy for reliable clinical decision-making are just a few of the significant obstacles these systems still must overcome [97].

Different physiological and analytical challenges are introduced by each alternate biofluid. Sweat rate, skin contamination, evaporation, temperature, pH, electrolyte composition, and local gland activity all have a significant impact on sweat glucose, which is frequently present at substantially lower concentrations than blood glucose. Urine glucose is not appropriate for fine-scale, real-time glycemic surveillance because it reflects renal glucose processing and typically appears only at substantial levels after blood glucose exceeds the renal threshold. Under certain circumstances, tear turnover, evaporation, ocular discomfort, lens movement, and fluctuations in tear-film composition can all affect the relationship between tear glucose and blood glucose. Additional difficulties with saliva-based glucose detection [33] include discomfort or inconsistent sample collection, possible filtration or dilution requirements, low biomarker concentration, enzymatic degradation, food contamination, oral microbiome effects, pH variation, and significant compositional variability. These factors make it more difficult to develop reliable saliva-based glucose monitoring systems and reduce the quantitative correlation between salivary and blood glucose levels.

The limitations of blood- and alternative-biofluid sensors have intensified interest in optical methods that avoid direct analyte consumption. These approaches offer repeated interrogation but introduce a different set of signal-generation and tissue-interference challenges. Despite these obstacles, alternative glucose detection techniques are still developing, with optical glucose-monitoring technology among the most promising avenues for continuous, non-invasive monitoring. The potential for non-destructive, reagent-free, or minimally reagent-dependent glucose detection makes optical glucose-monitoring techniques, especially Raman spectroscopy [18] and fluorescence [71], appealing. Optical methods may provide real-time monitoring, repeated interrogation, and extended sensor lifetimes, since they do not always consume glucose molecules during measurement, unlike many electrochemical sensors. Additionally, optical techniques can yield molecular, spatial, or spectroscopic data for multiplexed biochemical investigation, integration with wearable photonic systems, and enhanced specificity. Table 4 summarizes representative analytical performance metrics reported for major glucose-monitoring platforms, including detection limits, sensitivity, working ranges, biological matrices, and defining technical features.

However, biological interference and basic signal-generation limitations continue to impede optical glucose monitoring. Although glucose has a modest Raman scattering cross-section and its spectral characteristics are often masked by stronger signals from water, proteins, lipids, and other tissue constituents, Raman spectroscopy provides high molecular specificity. By using plasmonic nanostructures to amplify Raman signals, surface-enhanced Raman spectroscopy can increase sensitivity. However, this improvement comes with trade-offs concerning substrate reproducibility, hot-spot stability, long-term sensor lifespan, surface fouling, calibration transferability, and potential biotoxicity of metallic nanostructures or functional coatings [18,75]. Although optical coherence tomography [101,104] and photoacoustic spectroscopy [113] have demonstrated promise in vivo, their sensitivity in physiological settings remains insufficient for reliable glucose measurement. In these techniques, the effects of tissue temperature, hydration, perfusion, scattering, motility, and other metabolic components frequently outweigh the effects of glucose-induced optical or sonic alterations.

Similar restrictions apply to near-infrared and related optical spectroscopy techniques. The glucose absorption bands in the NIR range are weak, broad, and highly overlapped with those of water, hemoglobin, lipids, collagen, and other tissue absorbers, despite their appeal for non-invasive continuous glucose monitoring. As a result, a complex convolution of glucose concentration, tissue architecture, skin color, moisture, blood flow, temperature, probe pressure, and motion artifacts is frequently reflected in observed spectra. Practical implementation is further constrained by high sensor costs, limited miniaturization, calibration drift, and the requirement for customized calibration models. Therefore, despite the strong theoretical and technological appeal of optical approaches, significant advances in signal enhancement, physiological noise reduction, calibration robustness, and validation across diverse patient groups are necessary for their practical application.

All things considered, every technology has distinct trade-offs. Enzyme instability, consumable costs, biofouling, and invasiveness are the main drawbacks of enzymatic electrochemical sensors, notwithstanding their superior specificity and commercial maturity. Although they offer better chemical and thermal resistance, non-enzymatic electrochemical sensors often exhibit inadequate selectivity in complex biofluids. Although wearable alternative-biofluid sensors improve accessibility and comfort, they face challenges due to matrix interference, low glucose concentrations, inconsistent sampling rates, and poor blood correlation. Although optical technologies offer the potential for painless, continuous monitoring, they must overcome tissue heterogeneity, motion sensitivity, weak glucose-specific signals, high apparatus costs, and calibration instability. Although microwave and radiofrequency methods are appealing for wearable integration, they are nevertheless susceptible to dielectric interference from temperature, water, electrolytes, and tissue thickness.

In summary, despite significant advancements, there remain substantial scientific, technological, and translational obstacles to existing glucose-monitoring systems. For next-generation non-invasive monitoring, fluorescence sensing, Raman spectroscopy, optical coherence tomography, and other optical techniques offer substantial benefits. However, there are still issues with maintaining signal strength, sensor longevity, probe biocompatibility, suppressing biological signal interference, and achieving clinically acceptable accuracy in real-world settings. Future developments in materials chemistry, biointerface engineering, nanophotonics, flexible electronics, microfluidics, antifouling coatings, multimodal sensing, and artificial intelligence-assisted calibration will be necessary. To enable dependable, painless, continuous, and accessible glucose monitoring for precision diabetes care, the most effective next-generation glucose-monitoring platforms will likely integrate robust transduction mechanisms with physiologically informed algorithms and patient-specific calibration techniques. Beyond analytical sensitivity, the clinical translation of glucose-monitoring technologies depends on long-term stability, biological compatibility, calibration robustness, and real-world usability. Table 5 summarizes the principal translational bottlenecks and future engineering priorities for each technology class. These unresolved issues show that the next major advance will not arise from sensitivity enhancement alone. Successful translation will require coordinated progress in sensing chemistry, biointerface design, clinical validation, affordability, regulation, and computational interpretation.

## 8. Clinical Performance Requirements for Truly Non-Invasive Glucose Monitoring

Before a truly non-invasive glucose-monitoring system can replace established CGM or finger-prick testing, it must demonstrate performance using clinically meaningful criteria rather than analytical sensitivity or correlation alone. First, the device should provide sufficiently accurate glucose estimation across the clinically relevant glycemic range, including normoglycemia, hyperglycemia, and, critically, hypoglycemia. Quantify agreement with an accepted reference measurement using appropriate error metrics such as mean absolute relative difference (MARD), mean absolute difference, bias, precision, and limits of agreement, and clearly define the selected metric. Clinical acceptability should also be evaluated using established error-grid approaches, such as the Clarke or Parkes error grid, to determine whether measurement errors are likely to result in clinically inappropriate treatment decisions.

For continuous or near-continuous systems, performance should also include temporal characteristics such as response time, physiological lag, trend accuracy, and the ability to identify clinically meaningful rates of glucose change and hypoglycemic or hyperglycemic excursions. A candidate non-invasive sensor should maintain performance under realistic physiological and environmental conditions, including movement, exercise, perspiration, hydration changes, temperature variation, tissue thickness, skin pigmentation, pressure, and changes in peripheral perfusion. Demonstrate long-term stability and repeatability over clinically relevant periods, not just during short laboratory experiments, and quantify the need for recalibration. Ideally, a replacement technology should minimize or eliminate dependence on intermittent finger-prick calibration.

Clinical validation should use sufficiently large and independent patient cohorts representing different ages, diabetes types, glycemic ranges, comorbidities, skin characteristics, and daily activity conditions. Prospective validation against an appropriate reference method is particularly important for distinguishing a generalizable clinical technology from a device whose performance depends on a limited laboratory dataset. Finally, replacement of conventional CGM or finger-prick testing requires consideration of patient comfort, safety, wearability, reliability, affordability, data integrity, and usability in routine daily life. Thus, a truly non-invasive glucose monitor should be considered clinically successful only when it combines acceptable glucose-estimation error with robust longitudinal performance, safe operation, low user burden, and reproducible performance across diverse real-world populations.

## 9. Conclusions, Translational Outlook, and Future Hope Perspectives

Glucose monitoring is a central component of diabetes management [133]. It supports timely therapeutic intervention and helps patients maintain glucose within individualized target ranges. Effective monitoring can reduce acute hypo- and hyperglycemic events and lower the risk of long-term vascular, neurological, renal, and ocular complications [136]. Glucose-monitoring technology has changed significantly over the last several decades, moving from laboratory-based assays and sporadic finger-prick testing to wearable, continuous, minimally invasive, and progressively non-invasive sensing platforms. This development is indicative of a broader paradigm shift in the treatment of diabetes, with real-time, predictive, and personalized metabolic control replacing retrospective and episodic glucose testing.

To reduce dependence on inserted electrochemical probes, optical and electromagnetic approaches seek to recover glucose information without direct tissue penetration. A wide range of glucose-monitoring modalities, such as optical [106], colorimetric [55], near-infrared [110], photoacoustic [113], and related technologies, have been made possible by recent developments in materials engineering, nanotechnology, microfabrication, flexible and stretchable electronics, photonics, wireless communication, and computational analytics. The potential for non-invasive or user-friendly continuous monitoring is enhanced by several of these methods, which also enable painless or less uncomfortable sampling from other biofluids, such as tears [129], saliva [33], and perspiration [95]. Smart contact lenses [70], wearable sensors [88], and epidermal patches [88] are examples of emerging device types that aim to provide real-time glucose information while enhancing patient adherence, comfort, and mobility. Taken as a whole, these developments indicate that the field is moving toward decentralized glucose surveillance systems capable of operating continuously outside regulated clinical settings.

Due to their straightforward working principle, quick reaction, small instrumentation, low power consumption, scalable production, and high prediction accuracy, electrochemical glucose sensors continue to be the most extensively used technology [41]. Enzyme-mediated specificity, an established strip-based testing infrastructure, and compatibility with smaller wearable devices have all contributed significantly to their clinical success. Enzyme instability, electrode fouling, chemical interference, oxygen dependency, calibration drift, material biocompatibility, and limited long-term operational resilience are some of the basic and practical difficulties that electrochemical sensors currently confront despite their widespread usage [15]. These difficulties are especially noticeable in continuous-monitoring settings, where sensors must withstand biofouling, inflammation, mechanical deformation, and shifting local tissue chemistry while operating for prolonged periods of time in dynamic biological media.

Ion-selective field-effect transistors (ISFETs) [121,122,123,130] and extended-gate field-effect transistors (EGFETs) [32] are two examples of transistor-based glucose sensors that offer an appealing path toward quick, label-free, compact, and semiconductor-compatible glucose detection. These platforms enable quick data capture and repeatable device topologies by converting biological interactions at the gate interface into variations in surface potential, threshold voltage, or channel current. However, low sensitivity in certain designs, electrical noise, ionic screening, baseline drift, gate dielectric instability, nonspecific adsorption, and interference from complex physiological matrices continue to limit their broader application. Advanced gate-interface engineering, low-noise circuit design, robust dielectric passivation, antifouling surface chemistries, and nanostructured functional layers that enhance glucose-dependent interfacial signals are all necessary for future advances in ISFET and EGFET glucose sensing.

Significant research has shifted toward optical glucose-monitoring platforms, such as fluorescence [17], Raman spectroscopy, optical coherence tomography [125], photoacoustic spectroscopy [128], and other spectroscopic techniques, to mitigate the drawbacks of invasive and semi-invasive monitoring. Because fluorescence-based sensing uses ultraviolet or visible LED excitation sources to enable sensitive, visually accessible, and perhaps inexpensive glucose monitoring, it has demonstrated great promise [53,54,55]. The molecular design space for glucose-responsive optical sensing has been extended by fluorescent probes, boronic acid receptors, quantum dots, hydrogels, carbon-based nanomaterials, and FRET-based structures. However, advances in photostability, suppression of autofluorescence, quantitative signal calibration, tissue penetration depth, probe biocompatibility, and resistance to photobleaching in long-term monitoring settings are still needed for fluorescence techniques.

Raman spectroscopy is appealing for glucose detection in complicated biological contexts because it provides great chemical specificity and clear molecular fingerprints [12,13,14]. By adding plasmonic substrates with strong electromagnetic hot spots, surface-enhanced Raman spectroscopy may greatly increase its intrinsically weak scattering signal [18,74,87]. Thus, SERS-based sensors offer a potent means of providing targeted metabolic monitoring and magnifying glucose signals. Nevertheless, substrate reproducibility, hot-spot heterogeneity, signal drift, surface fouling, limited sensor lifetime, potential nanomaterial biotoxicity, and difficulties in keeping glucose molecules within the effective near-field enhancement region continue to limit clinically reliable SERS glucose sensing. Reproducible nanofabrication, biocompatible plasmonic materials, self-referencing spectral methods, antifouling coatings, and reliable calibration frameworks will all be necessary to address these problems.

Since reverse iontophoresis removes glucose-containing fluid from the skin without requiring a traditional blood sample, it is another crucial method for real-time glucose monitoring with a lower risk of infection [91]. Low glucose concentrations in the retrieved interstitial fluid, variations in skin permeability, sweat contamination, electrochemical interference, skin irritation, and susceptibility to local hydration, temperature, and physiological state are among the technique’s limitations. Optical coherence tomography may identify changes in optical scattering and refractive index associated with glucose and offers depth-resolved, high-resolution, real-time monitoring with significant tissue penetration [125]. However, motion artifacts, contact pressure, tissue heterogeneity, temperature, humidity, and other physiological or environmental variables can significantly affect OCT-based glucose monitoring. Similarly, infrared absorption spectroscopy enables non-invasive examination of deeper tissue layers; however, it is significantly affected by various skin variables, such as temperature, perspiration, thickness, pigmentation, and humidity [107,108]. By combining optical absorption with acoustic detection, photoacoustic spectroscopy provides improved contrast; nonetheless, motion artifacts, ambient factors, tissue background signals, and sensitivity constraints continue to limit its practical application [128]. Although microwave-based glucose sensors have also demonstrated encouraging results for real-time monitoring on flexible substrates, their clinical applicability is still constrained by inadequate validation in realistic physiological settings, tissue-thickness variability, dielectric interference from water and electrolytes, and insufficient precision [114].

Taken together, the examined technologies show that there is not yet a single sensing modality that meets all the needs of optimal glucose monitoring. Although electrochemical sensors are mature and sensitive, they still have long-term stability issues and largely rely on invasive or minimally invasive sampling. Although weak glucose-specific signals, biological background interference, motion artifacts, and calibration instability must be addressed, optical technologies offer a promising path toward painless, non-invasive assessment. Although alternative biofluid sensors are more accessible and comfortable, they are constrained by low analyte concentrations, matrix variability, sampling uncertainty, and poor correlation with blood glucose. Although they are appealing for wearable integration, microwave and radiofrequency systems need significant improvements in robustness, specificity, and accuracy. Therefore, multimodal systems that integrate complementary transduction mechanisms, sophisticated biointerfaces, and adaptive computational models are likely to determine the future of glucose monitoring rather than a single dominant technique.

Future studies should focus on a number of interrelated goals. Higher sensitivity, selectivity, and stability under physiologically relevant settings are the first requirements for sensing interfaces. Advanced nanomaterials, biocompatible encapsulating layers, antifouling coatings, enzyme-stabilization techniques, non-enzymatic catalytic surfaces, and mechanically compliant substrates will all be needed for this. Second, long-term wearability, repeatable skin or tissue contact, minimal discomfort, low power consumption, and wireless data transfer must all be considered when designing devices. Third, adaptive, patient-specific models that account for physiological lag, biofluid transport kinetics, tissue characteristics, environmental changes, and sensor aging must replace static, population-level calibration adjustments. Fourth, broad, diverse, real-world cohorts with varying ages, diabetes types, skin tones, comorbidities, exercise levels, medications, and glycemic ranges, particularly hypoglycemia cases, when accuracy is most crucial, must be included in clinical validation, in addition to small controlled trials.

One especially revolutionary path is the combination of machine learning, artificial intelligence, and glucose monitoring [128]. By learning from past sensor data, reference glucose levels, physiological context, and real-time feedback, AI-driven systems can enhance calibration. Sensor drift, temperature fluctuations, motion artifacts, perspiration interference, optical scattering variations, radiofrequency noise, and other confounding factors that frequently reduce the accuracy of glucose sensors may all be dynamically compensated for using these models. In complicated physiological conditions where conventional calibration models are inadequate, machine-learning algorithms can also detect nonlinear correlations between raw sensor inputs and glucose levels, boosting prediction performance [111,134].

Regardless of the underlying transduction mechanism, calibration and physiological variability remain shared limitations. AI and machine learning may help address these challenges by integrating sensor outputs with contextual physiological data. The integration of multimodal data streams, such as glucose readings, heart rate, physical activity, sleep patterns, food intake, insulin dosage, stress indicators, skin temperature, hydration status, and other wearable-derived physiological signals, is made possible by AI in addition to calibration. Individualized treatment suggestions, early-warning alarms, and tailored glucose forecasts can all be supported by such integration. For instance, wrist-worn AI-powered systems that monitor changes in microwave resonance have shown remarkable accuracy in predicting glucose levels [119,134]. These devices enhance trend prediction, estimate glucose concentration, and calibrate real-time resonance data using machine learning algorithms. AI models may be improved to lower false alarms, enhance hypoglycemia prediction, and facilitate individualized diabetes care as datasets grow in size and representativeness.

However, incorporating AI into glucose monitoring requires careful consideration. The quality of the dataset, population diversity, sensor reliability, reference accuracy, and validation design all significantly impact algorithmic performance. When applied to diverse populations or real-world scenarios, models trained on small or uniform datasets may not perform well. Thus, explainability, uncertainty quantification, prospective validation, regulatory transparency, cybersecurity, data privacy, and clinically interpretable outputs should be prioritized in future AI-enabled glucose-monitoring systems. The objective should be to produce reliable, useful information that patients and physicians can safely utilize for diabetes care, rather than just improving numerical prediction.

An important methodological consideration in AI/ML-based glucose monitoring is whether training, validation, and test datasets are separated at the participant level. Randomly dividing individual measurements from the same participant across training and testing sets can cause information leakage because the model may learn subject-specific physiological characteristics, sensor responses, tissue properties, and repeated-measurement patterns that then reappear in the nominal test set. Such an approach may therefore produce performance estimates that are more optimistic than would be expected when the system is applied to previously unseen patients. Accordingly, AI-enabled glucose-monitoring studies should preferably use participant-level separation, in which all measurements from a given participant are assigned exclusively to the training, validation, or independent test cohort. External validation using previously unseen participants or, ideally, an independent clinical cohort provides stronger evidence of generalizability. Within the studies reviewed here, therefore, the validation strategy must be interpreted alongside the reported performance metrics. For example, the NIR/SDNN study by Srichan et al. used 401 blood samples for model development with ten-fold validation and then evaluated the model on a separate cohort of 234 individuals not included in model training [111]. This provides stronger participant-level separation for the external evaluation than random measurement-level splitting alone. By contrast, studies using random sampling across the complete dataset should be interpreted more cautiously when they do not explicitly demonstrate participant-level separation of repeated measurements. Future AI/ML glucose-monitoring studies should report the number of unique participants in each dataset, the number of measurements per participant, the data-splitting strategy, and whether any participant contributed data to more than one modeling subset.

Importantly, the future development of glucose-monitoring technologies should be evaluated not only according to analytical performance but also according to their impact on patient-centered outcomes, including comfort, affordability, usability, accessibility, and long-term adherence. Although CGM systems provide substantially richer temporal information than episodic glucose measurements, their widespread adoption in routine clinical care can be constrained by the cumulative cost of sensors, transmitters or readers, replacement components, calibration requirements, and associated digital infrastructure. These economic considerations may be particularly important for patients requiring long-term or intensive glucose monitoring and may contribute to disparities in access between different socioeconomic and healthcare settings. Future platforms should therefore prioritize low-cost sensing materials, scalable manufacturing, longer sensor lifetime, reduced dependence on disposable components, simplified calibration, and interoperable smartphone-based or point-of-care readout systems. Such approaches could reduce the overall cost of monitoring while preserving clinically meaningful accuracy and reliability. At the same time, AI-assisted calibration and predictive algorithms should be developed with attention to robustness across diverse patient populations and real-world physiological conditions, rather than being optimized only for controlled datasets. Ultimately, the successful translation of next-generation CGM and closed-loop systems will depend on achieving an appropriate balance between sensing accuracy, continuous data availability, patient comfort, affordability, usability, and equitable access. The goal should therefore be not simply to develop more intelligent glucose sensors, but to create sustainable and accessible monitoring systems that improve diabetes self-management and clinical decision-making across diverse healthcare settings.

Taken together, these developments indicate that future glucose monitoring will depend on convergence rather than a single sensing modality. In conclusion, wearable, intelligent, painless, continuous, and patient-specific technologies are the future of next-generation glucose monitoring. Electrochemical sensors, fluorescence probes, Raman and SERS platforms, OCT, infrared spectroscopy, photoacoustic techniques, reverse iontophoresis, microwave sensing, smart contact lenses, epidermal patches, and wearables with AI capabilities have all significantly improved the discipline. However, achieving clinically accurate glucose monitoring in intricate, dynamic, and diverse biological settings remains the fundamental challenge. Deep integration of materials science, bioengineering, photonics, electronics, physiology, data science, and clinical medicine will be necessary to meet this challenge. Future glucose-monitoring platforms may evolve from passive sensors into adaptive metabolic-intelligence systems. These systems could combine glucose measurement with trajectory forecasting, decision support, and treatment guidance. Achieving this goal will require sustained interdisciplinary collaboration and rigorous clinical validation.

Ultimately, the clinical value of next-generation glucose-monitoring technologies will be determined not solely by their analytical sensitivity or algorithmic sophistication, but by their ability to deliver accurate, comfortable, affordable, reliable, and equitable monitoring that patients can sustain in routine daily life and that clinicians can meaningfully integrate into personalized diabetes care.

## Figures and Tables

**Table 1:** Secondary/Hybrid Transduction Strategy.

Technology Class	Invasiveness	Sampling	Primary Transduction	Secondary/Hybrid Component
AI wrist-worn microwave	Non-invasive	Tissue	Microwave/Radiofrequency	AI/ML
Smart contact lens	Non-invasive	Tears	Electrical/optical	Wireless power/display
SERS microneedle	Minimally invasive	Interstitial Fluid	Raman/SERS	Microneedle sampling
mbNIR + SDNN	Non-invasive	Tissue	NIR	AI/ML
OCT + 3D correlation	Non-invasive	Tissue	Subcutaneous interstitial fluid	Computational analysis

**Table 2:** Comparative Overview of Major Glucose-Monitoring Technologies According to Invasiveness, Sampling Matrix, and Transduction Strategy.

Technology Class	Representative Modality	Invasiveness Level	Primary Sampling Matrix/Site	Core Sensing Principle	Main Advantages	Principal Limitations	References
Conventional electrochemical glucose sensors	Enzymatic amperometric/potentiometric glucose sensors	Invasive	Capillary blood, interstitial fluid, skin-inserted sensors	Glucose oxidase/glucose dehydrogenase-mediated redox reaction converted into electrical current or potential	Simple device architecture, rapid response, low cost, high predictive accuracy, mature clinical use	Pain, invasiveness, infection risk, enzyme instability, interference, biofouling, biocompatibility concerns	[24,25,26,27,28,29,30]
Non-enzymatic electrochemical sensors	Metal, metal oxide, MOF, carbon, and conductive-polymer electrodes	Invasive/minimally invasive	Blood, interstitial fluid, sweat	Direct electrocatalytic oxidation of glucose at nanostructured electrode surfaces	Improved chemical/thermal stability, enzyme-free operation, high sensitivity possible	Selectivity challenges in complex biofluids, surface fouling, reproducibility issues, alkaline-condition dependence in some systems	[10,15,31,32,33,34,35,36,37,38,39,40,41,42,43]
Microdialysis-assisted electrochemical sensing	Implantable microfluidic microdialysis sensors	Invasive	Subcutaneous interstitial fluid	Diffusion of glucose through semipermeable dialysis membrane followed by electrochemical detection	Enables continuous sampling, reduces direct electrode–tissue contact, suitable for real-time tracking	Implantation discomfort, device complexity, delayed transport, calibration requirements	[23,42]
Hexokinase photometric assay	Hexokinase/glucose-6-phosphate dehydrogenase method	In vitro/reference method	Blood or biological sample	Glucose phosphorylation followed by NADH generation measured at 340 nm	High specificity, reference-quality quantification, strong biochemical reliability	Not wearable, requires reagents and optical instrumentation, enzyme instability	[23,44,45]
Field-effect transistor sensors	ISFET, EGFET, GFET, P-GFET	Invasive/minimally invasive/ex vivo	Interstitial fluid, saliva, tears, urine	Glucose-induced interfacial charge/potential changes modulate channel current, threshold voltage, or Dirac point	Fast response, miniaturizable, reusable, compatible with semiconductor platforms	Electrical noise, ionic screening, drift, low sensitivity in some designs, matrix interference	[32,33,34,46,47,48,49,120,121,122,123]
Fluorescence-based sensors	Boronic acid probes, Con A systems, FRET sensors, hydrogel probes	Minimally invasive/non-invasive	Interstitial fluid, tears, serum, implantable hydrogel environment	Glucose binding or enzymatic reaction modulates fluorescence intensity, lifetime, or ratiometric signal	High sensitivity, visual/optical readout, compatible with low-cost LEDs, potential long-term monitoring	Autofluorescence, photobleaching, skin/tissue scattering, pigmentation effects, probe stability	[16,17,50,51,52,53,54,55,56,57,58,59,60,61,62,63,64,65,66,67,68,69,70,71,72]
Raman/SERS sensors	Raman spectroscopy, SERS nanoprobes, plasmonic microneedles	Minimally invasive/non-invasive	Interstitial fluid, tumor spheroids, biological fluids, skin-related sites	Molecular vibrational fingerprinting enhanced by plasmonic hot spots	High chemical specificity, multiplexing potential, label-free molecular information	Weak native Raman signal, expensive instrumentation, calibration burden, SERS substrate variability, possible nanotoxicity	[12,13,14,15,16,17,18,19,20,21,22,51,73,74,75,76,77,78,79,124]
Microneedle-array glucose sensors	Hollow, coated, polymeric, SERS-active, electrochemical microneedles	Minimally invasive	Dermal/interstitial fluid	Microneedles access ISF and integrate electrochemical, optical, or theranostic sensing	Reduced pain, direct ISF access, wearable integration, potential continuous monitoring	Insertion-depth variability, skin irritation, biofouling, sterilization, mechanical durability	[52,83,84,85,86,87]
Reverse iontophoresis	Transdermal electro-osmotic glucose extraction	Non-invasive/minimally invasive hybrid	Extracted interstitial fluid, sweat, skin	Low electric current drives Na^+^-mediated electro-osmotic solvent flow carrying glucose across the skin	Low infection risk, real-time potential, compatible with flexible electronics	Low extracted glucose concentration, skin irritation, sweat interference, physiological lag, calibration complexity	[88,89,90,91,92,93,94,95,96,97,98,99]
Optical coherence tomography	OCT-based scattering measurement	Non-invasive	Skin/tissue	Glucose-induced refractive-index changes alter tissue scattering and OCT signal attenuation	Depth-resolved imaging, real-time measurement, high spatial resolution	Motion artifacts, skin temperature/humidity sensitivity, weak glucose-specific signal, high cost	[100,101,102,103,104,105,106,125,126,127]
Infrared absorption spectroscopy	NIR/mid-IR spectroscopy	Non-invasive	Skin/tissue	Glucose-related overtone/combination absorption bands from C–H and O–H vibrations	Deep tissue interrogation, non-contact potential, compatible with ML calibration	Weak spectral signatures, overlap with water/tissue absorbers, skin variability, calibration transfer issues	[103,106,107,108,109,110,111,112]
Photoacoustic spectroscopy	Laser-induced acoustic detection	Non-invasive	Skin, aqueous humor, tissue	Optical absorption produces thermoelastic expansion and acoustic waves	Contactless potential, improved sensitivity over some optical methods, water has weak PA response	Motion sensitivity, temperature/pressure interference, physiological background complexity	[103,128]
Microwave/radiofrequency sensors	Split-ring resonators, RF biosensors, dial-resonating sensors	Non-invasive	Skin/tissue, interstitial-fluid-related dielectric environment	Glucose-induced dielectric changes shift resonance frequency, phase, amplitude, or quality factor	Real-time monitoring potential, flexible substrates, passive sensing possible	Lower precision, dielectric interference from water/electrolytes/tissue thickness, limited clinical validation	[114,115,116,117,118,119]
Smart contact-lens sensors	Graphene/contact-lens glucose sensors with wireless power and LED display	Non-invasive	Tears	Tear glucose modulates sensor resistance and LED output through wireless circuit integration	Wearable ocular platform, real-time visualization, no bulky external instrumentation	Tear–blood correlation uncertainty, RF interference, ocular biocompatibility, sensor fouling, low signal-to-noise ratio	[70,118,129]
AI/ML-assisted glucose-monitoring systems	ML-calibrated NIR, microwave, optical and wearable systems	Cross-cutting	Multimodal physiological signals	Algorithms correct drift, noise, nonlinear tissue effects, and individual variability	Personalized calibration, trend prediction, improved accuracy, multimodal integration	Requires large datasets, risk of overfitting, regulatory complexity, explainability and data privacy issues	[111,112,119,130]

**Table 3:** Representative Commercial Continuous Glucose Monitoring Systems and Key Clinical Characteristics.

System	Manufacturer	Sampling	Wear Duration	Calibration	Key Strengths	Key Limitations
FreeStyle Libre	Abbott	ISF	manufacturer-specific	factory calibrated for many versions	accessibility, flash/CGM ecosystem	interstitial lag, cost
Dexcom G6/G7	Dexcom	ISF	manufacturer-specific	factory calibrated	alerts, integration	cost, insertion
Medtronic Guardian	Medtronic	ISF	manufacturer-specific	system-dependent	pump integration	calibration/wear considerations
Eversense	Senseonics	ISF	long-term implantable	system-dependent	long wear duration	implantation procedure

**Table 4:** Analytical Performance Metrics of Representative Glucose-Monitoring Platforms Reported in Recent Studies.

Technology/Platform	Representative Device/Material	Detection Range or Working Range	Reported Sensitivity/Detection Limit/Performance	Measurement Site/Sample	Key Technical Feature	References
Non-enzymatic electrochemical sensor	Gold nanowire array electrode	0.5–20 mM glucose	Detection limit: 30 μM; sensitivity: 41.9 μA mM^−1^ cm^−2^	Electrochemical solution/in vitro	Direct partial oxidation of glucose on Au nanowire array	[31]
Stretchable Au nanowire electrochemical electrode	Enokitake mushroom-shaped Au nanowire films	0–800 μM	Sensitivity: 4.55 μA mM^−1^ cm^−2^ under 30% strain	Wearable/sweat-oriented platform	Stretchable nanowire morphology for strain-tolerant sensing	[36]
MOF-based non-enzymatic sensor	Co-MOF nanosheet array on nickel foam	Noted for high-performance non-enzymatic glucose detection	Detection limit: 1.3 nM; sensitivity: 10,886 μA cm^−2^ mM^−1^	Electrochemical/in vitro	High-porosity MOF with abundant catalytic sites	[10]
Conductive-polymer hybrid sensor	Hollow CuO/PANI nanofibers	0.001–19.899 mM	Detection limit: 0.45 μM	Electrochemical/in vitro	Coupling of CuO catalysis with conductive PANI network	[41]
Microdialysis-based microfluidic sensor	Pt–Ni NPs–MWCNTs/SPE integrated with microdialysis channel	Continuous microdialysis sampling	Detection limit: 21 μM	Subcutaneous ISF-oriented microfluidic platform	Microdialysis extraction combined with non-enzymatic electrochemical readout	[42]
Fiber-based wearable electrochemical sensor	Helical gold-fiber sweat sensor	0–200% strain tolerance	Sensitivity: 13.9 μA mM^−1^ cm^−2^	Sweat	Strain-insensitive electrochemical behavior using stretchable gold fibers	[43]
Hexokinase photometric method	HK/G6PDH enzymatic assay	Reference biochemical range	Absorbance-based NADH quantification at 340 nm; commonly detects clinically relevant glucose levels	In vitro sample analysis	Stoichiometric NADH generation proportional to glucose concentration	[23,44,45]
Graphene FET enzymatic biosensor	Silk fibroin-encapsulated graphene FET	0.1–10 mM	Detection limit: 0.1 mM	In vitro	Dirac-point/current modulation through GOx immobilized in silk fibroin	[34]
TiO_2_/Ti EGFET sensor	GOx-containing silk fibroin membrane on TiO_2_/Ti gate	Low-concentration glucose detection	Detection limit: 0.001 mg/mL	Biosensing interface	Extended-gate FET with enzyme-containing silk fibroin membrane	[32]
ZnO nanoarray EGFET	ZnO nanoarray extended-gate electrode	20–100 μM	Sensitivity: −0.395 mV/μM; response time: 6–7 s	Low-glucose biofluids such as saliva/tears	High-specific-surface-area ZnO nanoarray	[48]
Polymer-functionalized GFET	AAPBA-based polymer-functionalized graphene FET	0.04–10 mM	Detection limit: 1.9 μM; sensitivity: 822 μA cm^−2^ mM^−1^; stable over 20 cycles	Human urine/ex vivo	Boronic-acid–glucose covalent interaction modulates Dirac point/current	[49]
Fluorescence hydrogel glucose sensor	Boronic-acid hydrogel protected by SOD and catalase	Long-term in vivo monitoring	Stable fluorescence response for >28 days	In vivo implantable hydrogel environment	Antioxidant-enzyme protection of arylboronic acid from H_2_O_2_ degradation	[63]
Commercial fluorescent chemosensor platform	Modified diboronic acid fluorescent chemosensor/Senseonics Eversense concept	Long-term CGM	Continuous monitoring up to 365 days with a single sensor	Subcutaneous sensor	Fluorescence chemosensor integrated with algorithmic correction	[68]
SERS glucose sensor	Mixed decanethiol/mercaptohexanol Ag substrate	Physiologically relevant glucose measurement	Detection limit: 0.55 mM	Bovine plasma/in vitro	Partition layer enriches glucose near SERS-active surface	[51]
SERS-gNPS nanosensor	SERS glucose nanoparticle sensor	1–10 mM	Detection limit: 0.1 mM	3D tumor spheroids/in vitro	Continuous intratumoral glucose-gradient monitoring over 5 days	[18]
Microneedle-based CGM	Hollow microneedle with integrated electrochemical probe	Up to 14 mM	Sensitivity: 1.5 nA/mM	Dermal interstitial fluid/in vivo human testing	Electrochemical probe integrated inside hollow microneedle lumen	[52]
Smartphone-controlled microneedle CGM	Hollow microneedle wearable CGM system	In vitro glucose monitoring	RSD: 4.8%	ISF-oriented wearable platform	Smartphone-controlled device with flexible sensing electrodes	[84]
Integrated wearable microneedle array	Disposable microneedle sensor + reusable electronics	Continuous multi-biomarker monitoring	Wireless real-time glucose/lactate monitoring	Interstitial fluid	Replaceable disposable sensor with reusable electronics	[85]
Reverse iontophoresis/sweat platform	Electrochemically enhanced iontophoresis interface	Sweat analyte monitoring	Sensitivity: 2.1 nA/μM for glucose	Sweat	Integrated sweat induction and sensing on flexible platform	[95]
Touch-actuated RI glucose sensor	Microneedle array + RI + electrochemical sensor	ISF extraction/detection	Sensitivity: 7.76 μA mM^−1^	Interstitial fluid	Combined shallow penetration, RI extraction, and electrochemical detection	[96]
OCT glucose monitor	OCT-based non-invasive glucose sensing	Tissue depth up to ~1.6 mm	In vivo correlation R = 0.88 in early studies; ARD 11.5% in pilot OCT device study	Skin/tissue	Scattering changes associated with glucose-dependent refractive-index variation	[101,102,103,104,126,127]
3D OCT correlation method	Wristband-style OCT probe	Non-invasive blood glucose estimation	88.4% of data points in Clarke error grid Zone A	Skin/tissue	3D correlation and exclusion of low-correlation scattering regions	[105]
NIR fiber-optic spectroscopy	Rat skin NIR fiber probe	90–630 mg/dL	SEP: 35.6 mg/dL	Rat skin/in vivo	NIR spectral response to glucose variation	[107]
Clinical NIR glucose sensor	NIR sensor in diabetic and healthy subjects	50–500 mg/dL	SEP: 27.2 mg/dL	Human skin/clinical	Calibration model for non-invasive glucose prediction	[109]
mbNIR + SDNN model	Multiple photonic band NIR with personalized medical features	60–400 mg/dL	Accuracy: 98.5%; precision: 98.0%; sensitivity: 96.2%; specificity: 99.3%	Human non-invasive monitoring	Personalized medical features integrated with shallow dense neural networks	[111]
Multiwavelength optical + ML model	Optical sensor with machine-learning calibration	Clinically relevant glucose prediction	99.75% of readings in clinically acceptable Clarke error grid zones	Non-invasive optical monitoring	ML-assisted calibration of multiwavelength optical data	[112]
Microwave resonator sensor	Chipless split-ring microwave resonator	Continuous-time monitoring concept	Non-invasive real-time response; lower precision noted	ISF-related dielectric environment	Passive substrate-free resonator tag electromagnetically coupled to reader	[114]
AI wrist-worn microwave device	Dial-resonating sensor with AI algorithm	Four-cohort pilot evaluation	Mean absolute relative difference: 10.3%	Wrist-worn non-invasive monitoring	AI-assisted interpretation of microwave resonance shifts	[119]

**Table 5:** Translational Barriers and Future Engineering Priorities for Next-Generation Glucose-Monitoring Systems.

Technology Category	Current Translational Bottlenecks	Biological/Physiological Confounders	Device-Level Engineering Needs	Algorithmic/Computational Needs	Future Development Priority	References
Enzymatic electrochemical sensors	Enzyme degradation, calibration drift, disposable-strip cost, limited long-term stability	pH, temperature, humidity, oxygen availability, electroactive interferents, biofouling	More stable enzyme immobilization, antifouling coatings, mediator optimization, flexible electrode integration	Drift correction, adaptive calibration, interference compensation	Long-lifetime enzymatic interfaces with reduced calibration burden	[24,25,26,27,28,29,30,134]
Non-enzymatic electrochemical sensors	Selectivity limitations, surface fouling, reproducibility of nanostructured electrodes	Interfering molecules in blood/sweat/ISF, protein adsorption, ionic strength variation	Rational catalytic surface design, hybrid nanomaterials, stable porous electrodes, improved biocompatibility	Signal deconvolution and matrix-specific calibration	Clinically selective non-enzymatic sensors operating in physiological media	[10,15,31,32,33,34,35,36,37,38,39,40,41,42,43]
Microdialysis-based systems	Implantation burden, microfluidic complexity, delayed sampling, device maintenance	ISF transport kinetics, membrane fouling, local tissue response	Miniaturized microfluidics, low-fouling membranes, integrated pumps/sensors	Lag correction and dynamic ISF-to-blood conversion	Fully integrated microdialysis–sensor systems with reduced invasiveness	[23,42,98]
Field-effect transistor sensors	Electrical noise, drift, ionic screening, gate instability, matrix interference	High ionic strength of biofluids, nonspecific adsorption, pH fluctuation	Stable gate dielectrics, nanostructured gates, antifouling functionalization, low-noise readout circuits	Baseline correction, drift modeling, multi-parameter compensation	Robust solid-state biosensors for low-volume biofluids	[32,33,34,46,47,48,49,120]
Fluorescence sensors	Photobleaching, autofluorescence, probe degradation, limited tissue penetration	Skin pigmentation, tissue scattering, inflammatory encapsulation, optical attenuation	Photostable fluorophores, NIR/second-NIR probes, hydrogel encapsulation, implantable optics	Ratiometric/lifetime-based calibration, optical path-length correction	Long-term implantable or wearable fluorescence sensors with self-referenced output	[16,17,50,51,52,53,54,55,56,57,58,59,60,61,62,63,64,65,66,67,68,69,70,71,72,135]
Raman/SERS sensors	Weak native Raman signals, expensive instrumentation, SERS substrate variability, limited reproducibility	Tissue background spectra, molecular adsorption variability, biofouling	Reproducible plasmonic substrates, biocompatible nanostructures, miniaturized Raman optics	Spectral deconvolution, internal standards, ML-assisted peak extraction	Clinically reproducible SERS platforms with stable hot-spot engineering	[12,13,14,15,16,17,18,19,20,21,22,51,73,74,75,76,77,78,79,124]
Microneedle-array sensors	Skin irritation, insertion-depth variability, mechanical failure, sterilization, limited wear duration	Local inflammation, ISF composition variability, sweat contamination, tissue remodeling	Strong yet biocompatible microneedles, controlled insertion mechanics, integrated flexible electronics	Motion-artifact rejection, ISF-to-blood glucose modeling	Wearable microneedle CGM with standardized insertion and long-term skin compatibility	[52,83,84,85,86,87]
Reverse iontophoresis	Low extracted glucose concentration, skin irritation, poor hypoglycemia detection, calibration complexity	Skin hydration, sweat rate, temperature, permeability, microcirculation variability	Soft conformal electrodes, controlled current density, integrated sweat/temperature sensors	Physiological transport modeling, adaptive calibration, hypoglycemia-specific algorithms	Reliable transdermal extraction with clinically accurate blood-glucose reconstruction	[88,89,90,91,92,93,94,95,96,97,98,99]
OCT-based sensing	Weak glucose-specific scattering signal, motion artifacts, high component cost, prototype-stage maturity	Skin temperature, humidity, hydration, perfusion, tissue heterogeneity	Wristband-style stable probes, compact OCT engines, pressure-controlled interfaces	3D correlation, motion correction, feature selection, personalized calibration	Portable OCT systems with validated real-world glucose accuracy	[100,101,102,103,104,105,106,132]
NIR/infrared spectroscopy	Weak and overlapping glucose absorption bands, poor calibration transferability, no broad commercial adoption	Water, hemoglobin, lipids, collagen, melanin, skin thickness, sweating, tattoos	Multiband/hyperspectral optics, high-SNR detectors, stable probe positioning	ML/AI calibration, personalized medical features, domain-generalizable models	Personalized, multispectral non-invasive sensors validated across diverse populations	[103,106,107,108,109,110,111,112]
Photoacoustic spectroscopy	Sensitivity limitations under physiological conditions, environmental and motion interference	Temperature, pressure, tissue optical absorption, acoustic coupling variability	Stable laser/acoustic modules, compact transducers, contactless probe optimization	Signal enhancement, physiological-noise correction, ML-based classification	Hybrid photoacoustic–ML platforms for stronger glucose-specific contrast	[113,133]
Microwave/RF sensors	Lower precision, dielectric interference, limited clinical validation	Water/electrolyte content, tissue thickness, temperature, motion, hydration	Flexible resonator design, stable antenna coupling, passive sensor tags	AI-assisted resonance interpretation, subject-specific calibration	Wearable microwave systems with robust dielectric compensation	[114,115,116,117,118,119]
Smart contact lenses	Tear–blood glucose correlation uncertainty, ocular safety, RF interference, fouling	Tear turnover, evaporation, blinking, ocular irritation, tear composition variability	Transparent stretchable electrodes, oxygen-permeable substrates, stable wireless power transfer	Threshold calibration, tear-to-blood conversion models, noise suppression	Safe, transparent, long-wear ocular glucose platforms with clinically validated tear correlation	[70,118,131]
AI/ML-assisted glucose monitoring	Dataset bias, overfitting, lack of explainability, regulatory uncertainty	Population variability, activity, diet, medications, comorbidities, sensor aging	Multimodal wearable integration, secure data pipelines, standardized reference measurements	Explainable AI, uncertainty quantification, prospective validation, continuous learning	Adaptive metabolic intelligence systems for predictive and personalized diabetes care	[111,112,119,130]

## Data Availability

No new data were created or analyzed in this study. Data sharing is not applicable to this article.

## Glossary

The following terminologies are used in this manuscript:

**Standardized expression**	**Meaning**
Glucose sensing	Glucose monitoring, when describing molecular recognition or signal generation
Glucose monitoring	Glucose sensing, when describing a complete device or clinical system
Invasive technology	Intrusive technology
Non-invasive	Noninvasive
Minimally invasive	Minimally-invasive
Interstitial fluid sensor	Interstitial-fluid sensor
Blood glucose monitoring	Blood glucose-monitoring
Continuous glucose monitoring system	Continuous glucose-monitoring system
Glucose monitoring device	Glucose-monitoring device
Analytical or clinical performance	Sensor accuracy, when no metric is specified
Minimally invasive sensor, not non-invasive	Painless sensor that crosses the skin
Invasive or implantable sensor, not non-invasive	Implanted sensor
Hybrid minimally invasive platform, not strictly non-invasive	Microneedle plus reverse-iontophoresis system

## Abbreviations

The following abbreviations are used in this manuscript:

**Sr. No.**	**Short Form**	**Long Form**
1.	WHO	World Health Organization
2.	T1D	Type 1 diabetes
3.	T2D	Type 2 diabetes
4.	CGM	Continuous glucose monitoring
5.	BGC	Blood glucose concentration
6.	ISF	Interstitial fluid
7.	GOD	Glucose oxidase
8.	GOx	Glucose oxidase
9.	GDH	Glucose dehydrogenase
10.	H_2_O_2_	Hydrogen peroxide
11.	FAD	Flavin adenine dinucleotide
12.	FADH_2_	Reduced flavin adenine dinucleotide
13.	CAT	Catalase
14.	SCE	Saturated calomel electrode
15.	Ag/AgCl	Silver/silver chloride
16.	Pt	Platinum
17.	Au	Gold
18.	Ag	Silver
19.	CuO	Copper oxide
20.	ZnO	Zinc oxide
21.	TiO_2_	Titanium dioxide
22.	Ti	Titanium
23.	MOF	Metal–organic framework
24.	Co-MOF	Cobalt-based metal–organic framework
25.	CNT	Carbon nanotube
26.	MWCNT	Multiwalled carbon nanotube
27.	SPE	Screen-printed electrode
28.	GCE	Glassy carbon electrode
29.	Pt–Ni NPs	Platinum–nickel nanoparticles
30.	MWCNTs/SPE	Multiwalled carbon nanotubes/screen-printed electrode
31.	Pt–Ni NPs–MWCNTs/SPE	Platinum–nickel nanoparticles–multiwalled carbon nanotubes/screen-printed electrode
32.	FESEM	Field-emission scanning electron microscopy
33.	SEM	Scanning electron microscopy
34.	AFM	Atomic force microscopy
35.	CV	Cyclic voltammetry
36.	DPV	Differential pulse voltammetry
37.	PANI	Polyaniline
38.	PPy	Polypyrrole
39.	PPV	Poly(phenylene vinylene)
40.	PEDOT	Poly(3,4-ethylenedioxythiophene)
41.	Nb	Niobium
42.	Ru	Ruthenium
43.	HK	Hexokinase
44.	HKI	Hexokinase I
45.	HKII	Hexokinase II
46.	ATP	Adenosine triphosphate
47.	ADP	Adenosine diphosphate
48.	G6P	Glucose-6-phosphate
49.	NAD	Nicotinamide adenine dinucleotide
50.	NADH	Reduced nicotinamide adenine dinucleotide
51.	CHO	Chinese hamster ovary
52.	YFP	Yellow fluorescent protein
53.	CytoB	Cytochalasin B
54.	ISFET	Ion-selective field-effect transistor
55.	FET	Field-effect transistor
56.	EGFET	Extended-gate field-effect transistor
57.	GFET	Graphene field-effect transistor
58.	P-GFET	Polymer-functionalized graphene field-effect transistor
59.	Dex-AgNPs	Dextran-capped silver nanoparticles
60.	Con A	Concanavalin A
61.	AAPBA	3-Acrylamidophenylboronic acid
62.	AAm	Acrylamide
63.	DMAPAA	N,N-dimethylaminopropyl acrylamide
64.	CQDs	Carbon quantum dots
65.	CDs	Carbon dots
66.	QDs	Quantum dots
67.	ODB	o-Diaminobenzene
68.	FRET	Fluorescence resonance energy transfer
69.	AuNPs	Gold nanoparticles
70.	AgNPs	Silver nanoparticles
71.	SOD	Superoxide dismutase
72.	UV	Ultraviolet
73.	GO	Graphene oxide
74.	CD-GOx	Cyclodextrin-modified glucose oxidase
75.	Eu	Europium
76.	NIR	Near-infrared
77.	MIR	Mid-infrared
78.	SERS	Surface-enhanced Raman spectroscopy
79.	SAMs	Self-assembled monolayers
80.	4-MPBA	4-Mercaptophenylboronic acid
81.	DT	Decanethiol
82.	MH	Mercaptohexanol
83.	PMMA	Poly(methyl methacrylate)
84.	MN	Microneedle
85.	MA	Microneedle array
86.	RSD	Relative standard deviation
87.	RI	Reverse iontophoresis
88.	FDA	Food and Drug Administration
89.	FPCB	Flexible printed circuit board
90.	GEDP	Glucose electrochemical detection platform
91.	EBFC	Enzymatic biofuel cell
92.	PMS	Power management system
93.	OCT	Optical coherence tomography
94.	ARD	Absolute relative difference
95.	CEG	Clarke error grid
96.	SEP	Standard error of prediction
97.	GTT	Glucose tolerance test
98.	InGaAs	Indium gallium arsenide
99.	mbNIR	Multiple photonic band near-infrared
100.	NN	Neural network
101.	ANN	Artificial neural network
102.	SDNN	Shallow dense neural network
103.	PMF	Personalized medical feature
104.	PA	Photoacoustic
105.	RF	Radiofrequency
106.	DRS	Dial-resonating sensor
107.	ML	Machine learning
108.	AI	Artificial intelligence
109.	LED	Light-emitting diode
110.	AC	Alternating current
111.	DC	Direct current
112.	mNFs	Metal nanofibers
113.	CVD	Cardiovascular disease
114.	POC	Point-of-care
115.	PBS	Phosphate-buffered saline
116.	μM	Micromolar
117.	mM	Millimolar
118.	nM	Nanomolar
119.	mg/dL	Milligrams per deciliter
120.	mmol/L	Millimoles per liter
121.	cm^−1^	Reciprocal centimeter/wavenumber
122.	μA	Microampere
123.	nA	Nanoampere
124.	mV	Millivolt
125.	pH	Potential of hydrogen
